# Morphometrics of complex cell shapes: lobe contribution elliptic Fourier analysis (LOCO-EFA)

**DOI:** 10.1242/dev.156778

**Published:** 2018-03-15

**Authors:** Yara E. Sánchez-Corrales, Matthew Hartley, Jop van Rooij, Athanasius F.M. Marée, Verônica A. Grieneisen

**Affiliations:** 1 Computational and Systems Biology, John Innes Centre, Norwich NR4 7UH, UK; 2 Theoretical Biology/Bioinformatics, Dept. of Biology, Utrecht University, Padualaan 8, 3584 CH Utrecht, The Netherlands

**Keywords:** Cell shape, Cellular Potts model, Image analysis, Pavement cells, *Arabidopsis thaliana*, *Drosophila*

## Abstract

Quantifying cell morphology is fundamental to the statistical study of cell populations, and can help unravel mechanisms underlying cell and tissue morphogenesis. Current methods, however, require extensive human intervention, are highly parameter sensitive, or produce metrics that are difficult to interpret biologically. We therefore developed a method, lobe contribution elliptical Fourier analysis (LOCO-EFA), which generates from digitalised two-dimensional cell outlines meaningful descriptors that can be directly matched to morphological features. This is shown by studying well-defined geometric shapes as well as actual biological cells from plant and animal tissues. LOCO-EFA provides a tool to phenotype efficiently and objectively populations of cells, here demonstrated by applying it to the complex shaped pavement cells of *Arabidopsis thaliana* wild-type and *speechless* leaves, and *Drosophila* amnioserosa cells. To validate our method's applicability to large populations, we analysed computer-generated tissues. By controlling *in silico* cell shape, we explored the potential impact of cell packing on individual cell shape, quantifying through LOCO-EFA deviations between the specified shape of single cells in isolation and the resultant shape when they interact within a confluent tissue.

## Abstract (precis)

**Summary:** A novel method for automated and quantitative assessment of complex cell shapes, including features such as protrusion number and amplitude, is demonstrated for *Arabidopsis*, *Drosophila* and *in silico* cells.

## INTRODUCTION

Cell geometry has long fascinated biologists (Thompson, 1917). This interest is driven by a wide range of underlying scientific questions. For instance, cell shape changes can be linked to physiological responses of cells, such as membrane protrusions during apoptosis and migration (Charras and Paluch, 2008), and can underlie cell behaviour, such as chemotaxis (Driscoll et al., 2012; Keren et al., 2008). It plays a key role in tissue morphogenesis during development (Lecuit and Lenne, 2007; Sherrard et al., 2010) and in homeostasis (Marinari et al., 2012; Veeman and Smith, 2013). Cell shape influences intracellular processes such as microtubule organisation (Ambrose et al., 2011; Gomez et al., 2016) and stress patterns in plant epithelia (Sampathkumar et al., 2014); it indirectly positions the plane of cell division (Besson and Dumais, 2011; Minc et al., 2011) and can even determine how a flower attracts pollinators (Noda et al., 1994). Given the rich diversity of processes in which cell shape plays a decisive role, either actively or passively, cell morphometrics, the qualitative and quantitative study of cell shape characteristics, is becoming very important for developmental biology. In parallel, advances in imaging technology and software allow us to collect remarkable amounts of cell morphological data, which in turn calls for analytical tools to enable extracting meaningful cell shape information (Zhong et al., 2012). In stark contrast to the technological advances in imaging, there are relatively few automatic and quantitative tools available to analyse complex cell shapes (Ivakov and Persson, 2013; Ljosa et al., 2012; Rajaram et al., 2012). This gap reflects the non-trivial nature of this task: cell shape is often irregular and variable, making it very difficult to establish universal criteria encompassing cell geometry.

**Fig. 1.**
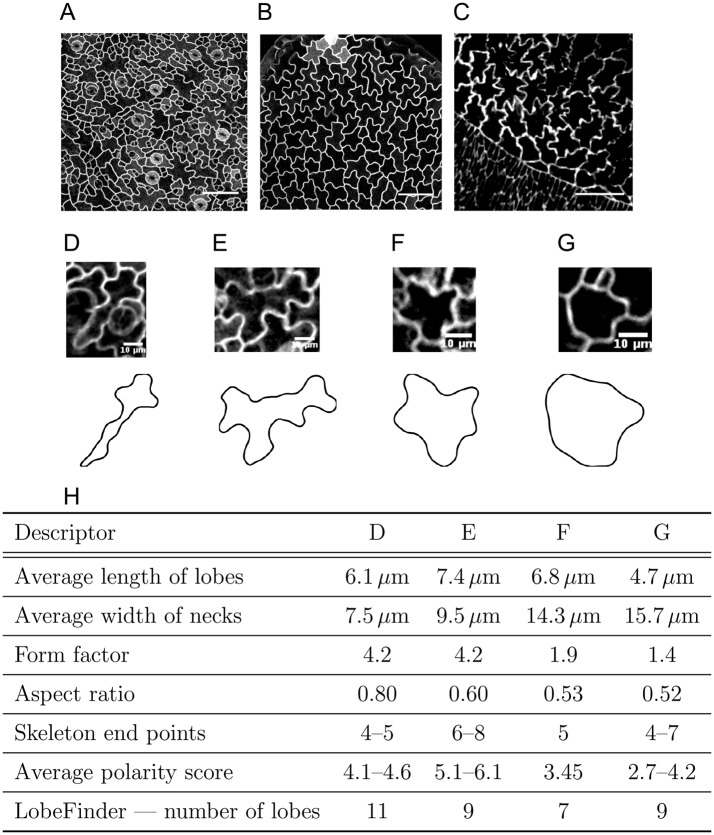
**Complex cell shapes and the shortcomings of traditional shape quantifiers.** (A-C) Complex cell shapes in both plant (A,B) and animal (C) tissues. (A,B) Pavement cells (PCs) of wild-type (A) and *speechless* mutant (B) *Arabidopsis thaliana* leaves, characterised by jigsaw-like shapes. (C) Amnioserosa cells in the *Drosophila* embryo present cell shapes with similar complexity. (D-G) Individual cells from the imaged tissues (upper panels), and the corresponding segmented cell outlines (lower panels). (H) Traditional metrics to quantify cell shape lead to similar values for very different shapes and are image-resolution and parameter sensitive. Here, the cells shown in D-G are compared. See also Fig. S1. Scale bars: 50 μm (A,B); 20 μm (C); 10 μm (D-G).

To illustrate the issues involved in quantitatively capturing complex cell shapes, we consider pavement cells (PCs) in the plant epidermis (Fig. 1A,B) and amnioserosa cells in the *Drosophila* embryo (Fig. 1C). PCs present a striking development, requiring multiple locally divergent growth fronts within each cell that are coordinated amongst neighbouring cells. Amnioserosa cells dynamically change their complex cell shape within a confluent tissue. Both cell types present challenges for quantifying cell shape: (1) their complex, non-holomorphic geometries cannot be captured in a meaningful way with traditional shape metrics; and (2) lack of recognisable landmarks excludes a myriad of shape analysis methods, such as Procrustes analysis (Klingenberg, 2010).

Traditional metrics for cell morphology include area, perimeter, aspect ratio and form factor. Although useful as general descriptors, they deliver limited shape information. Very different shapes may yield a similar aspect ratio or form factor (Fig. 1D-H). Besides not being unique, such descriptors tend to omit information regarding biologically relevant shape features. Several approaches to quantify complex cell shapes are summarised in Table 1. Some of these methods, such as the skeleton method, are highly sensitive to image noise as well as to the precise choice of parameters (for an example, see Le et al., 2006). Other metrics, such as lobe length and neck width (Fu et al., 2005), require humans to judge what a lobe is, which strongly impacts the quantitative results (Fig. 1, Fig. S1). It renders these metrics highly variable from cell to cell, from phenotype to phenotype and from human to human. To avoid such dependencies, an automatic method, LobeFinder, was developed to count lobes and indentations (Wu et al., 2016). This method, however, is less adapted to irregular cell shapes and estimation of lobe numbers using this method does not closely correspond to those defined by human inspection (Fig. 1). Moreover, it finds its limitations when the characteristics of a shape reside in the distribution and amplitude of the lobes, rather than in their number. For instance, some *Arabidopsis* mutants present PCs that are more elongated or have shallower lobes, but which occur at a similar spatial frequency (Lin et al., 2013). Recognising the need for automatic and non-biased quantification of PCs, Möller et al. (2017) developed PaCeQuant, a software to define lobes and necks in a systematic way based on local curvature. Similarly to LobeFinder, it is highly sensitive to small variations in the shape contour, with the sampling density of the contour biasing the local curvature estimation.

**Table 1.**
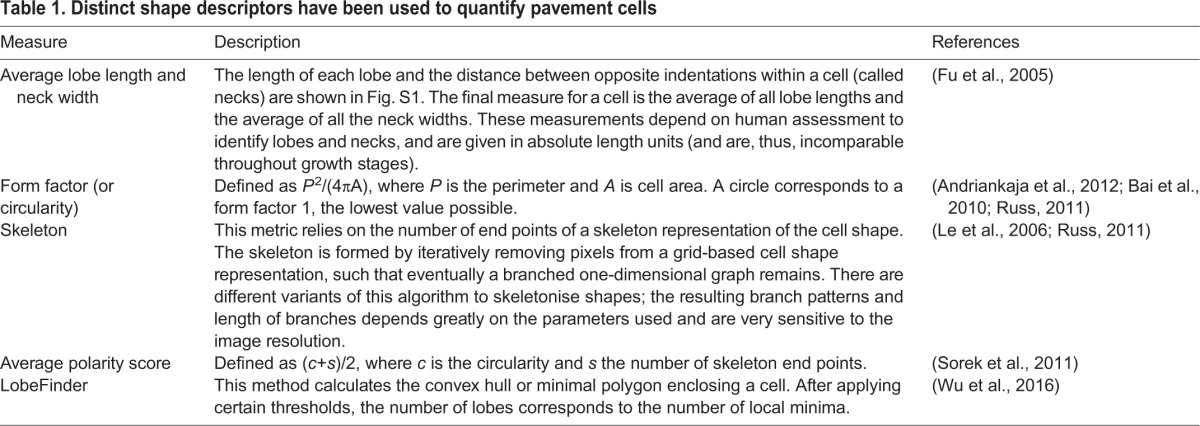
**Distinct shape descriptors have been used to quantify pavement cells**

Promising alternatives are methods that consider the full cell outline, reducing it into a series of coefficients that can be employed as shape descriptors in a multivariate study (Ivakov and Persson, 2013; Pincus and Theriot, 2007). Elliptical Fourier analysis (EFA) is such a method, used to quantify two-dimensional complex shapes (Diaz et al., 1989; Kuhl and Giardina, 1982; Schmittbuhl et al., 2003). In this method, the contour's coordinates are decomposed into a series of related ellipses (described by EFA coefficients), which can be combined to reconstitute the original shape. Despite its wide usage in morphometric studies, EFA cannot retrieve information that directly relates to morphological features of a cell, obstructing biological interpretation. This is because the same outline can be represented by infinitely many different sets of EFA coefficients, depending on how the cell outline is approximated, and because there is no one-to-one relationship between EFA modes and the number of morphological features (see supplementary Materials and Methods for further details).

Here, we present a new method based on EFA, termed lobe contribution elliptic Fourier analysis (LOCO-EFA), that overcomes the common obstacles described above. Our method also uses the whole two-dimensional cell contour but, unlike EFA, provides a set of metrics that directly relate to morphological features, permitting the assessment of cell shape complexity in an objective and automatic manner. Importantly, it is not sensitive to cell orientation or imaging resolution, and robustly yields similar coefficients for similar shapes, allowing shape comparisons to be drawn.

To validate the usage of our method on larger cellular datasets, we analyse confocal images of *Arabidopsis thaliana* PCs. We then complement this study with the analysis of synthetic tissues generated using the cellular Potts model (Glazier and Graner, 1993; Graner and Glazier, 1992), in which complex-shaped cells have a parametrised specified shape, allowing us to ask to what degree the resultant cell shape within a confluent tissue context is shaped by cell-to-cell interactions and to what degree it can be explained by intracellular shape control mechanisms. Applying LOCO-EFA to these abstract, *in silico* tissues (which rather mimic animal cells, with details regarding cell wall mechanics or chemical signalling not being considered), allows us to quantify the divergence of their specified cell shape when isolated to the shape taken up when immersed within a tissue. Finally, by applying LOCO-EFA to *Drosophila* data, we confirm its applicability to a wide range of biological systems.

## RESULTS

### Quantitative characterisation of cell shape using LOCO-EFA

Applying EFA to quantification of cell shapes, we came across a number of specific shortcomings. We first explain those issues to highlight our motivation and choices that led to the development of LOCO-EFA. See supplementary Materials and Methods for further details, such as mathematical implementation. Here, we focus on explaining the analysis in terms of its biological relevance, how it can be applied and interpreted.

The shape analysis proposed here is linked to frequency decompositions of digitalised two-dimensional shape outlines. We find it useful to compare the decomposition of a complex cell shape, such as that of a PC, to the way the sounds of musical instruments can be decomposed. When listening to a musical note, a quantifiable observable is the pitch. Within the context of PC shape, this corresponds to the observed number of lobes or, as we will explain in detail, to the dominant spatial frequency of the cell's outline. Another quantifiable property of a musical note is its volume, or amplitude. For cell shape, this corresponds to the extent to which lobes protrude and indentations retract, for which we also apply the term ‘amplitude’. Finally, the timbre of musical instruments is what essentially distinguishes, for example, a clarinet from an oboe playing the same note (pitch) at the same volume/amplitude. An analogous notion for cell shape studies is the ability to capture additional aspects of shape morphology that enable differences between cells to be quantified, even when the number of lobes (pitch) and their level of protrusion (amplitude) is the same.

**Fig. 2.**
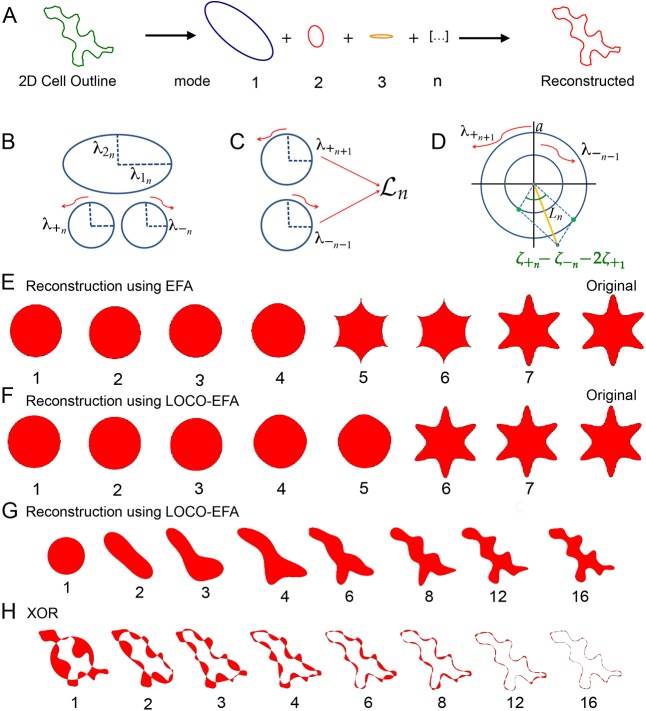
**LOCO-EFA retrieves correctly the cell shape's dominant spatial frequency.** (A) EFA decomposes a two-dimensional cell outline into an infinite summation of related ellipses or modes that can also be used to approximate the cell outline. (B) Each EFA harmonic is decomposed into two counter-rotating circles. (C) Mode 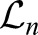 is composed of the counter-clockwise rotating *n*+1th harmonic circle and the clockwise rotating *n*−1th circle. (D) The combined amplitude contribution to *L_n_* (yellow line) of the two counter-rotating circles with radii 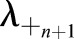 and 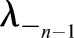 also depends on the offset in their starting points and the offset of the overall (mode 1) starting point, which together determine the initial phase shift (green dots) in the amplitude contribution of each rotor. (E,F) Comparison of closed contour reconstruction through either EFA (E) or LOCO-EFA (F). Although both approximations converge to the original six-lobed star shape (labelled ‘Original’), the reconstruction using EFA harmonics (E) generates a spurious shape after addition of the fifth harmonic and only recovers the original shape after the seventh harmonic, whereas the LOCO-EFA (F) reconstitutes the original shape precisely at the sixth mode, matching the protrusion number. The number of modes used for each sequential reconstruction is indicated below each shape. (G) LOCO-EFA reconstruction of a real cell taking the first *n* 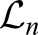 modes into account, as indicated below the panels. (H) Determination of the level of mismatch between the original cell shape and the *N*th mode truncated LOCO-EFA approximation, by applying the *XOR* (exclusive OR) function (see supplementary Materials and Methods).

As a starting point, EFA (Kuhl and Giardina, 1982) can describe the contour of any complex two-dimensional shape, including non-holomorphic shapes such as PCs, which most other methods are unable to handle (see Fig. S2 and supplementary Materials and Methods for further details). Using the coordinates of the two-dimensional outline (Fig. S3A), EFA decomposes the shape into an infinite series of ellipses (also referred to as ‘modes’ or ‘harmonics’, Fig. 2A). This series of ellipses, *n*=1…∞, can then be combined to retrieve the original shape exactly: each *n*th elliptic harmonic traces *n* revolutions around the first ellipse while orbiting around the previous (*n*−1) harmonic ellipse, which in turn orbits around its previous one (*n*−2), and so forth (Fig. S3B). This summation results in an outline being ‘drawn’, shown in Movie 1. A cut-off, *N*, sets the number of modes that are actually taken into account. In general, the value is determined for which the reconstituted cell contour is sufficiently close to the original outline (see further below).

The fact that each ellipse represents a harmonic suggests that it captures dominant spatial frequencies within the original shape. EFA harmonics have therefore been considered to be reasonable descriptors for shape (Schmittbuhl et al., 2003). However, the pitch, i.e. the most basic cellular feature to quantify, is actually not directly retrieved by EFA, even for simple shapes. For instance, a six-sided shape is expected to present a strong contribution from the sixth mode. Instead, EFA represents such a shape as a mixed contribution from the two adjacent modes, the fifth and seventh (Fig. 2E, Fig. S3C). This mismatch arises from how individual EFA modes contribute to the outline. When an outline is approximated, each elliptical mode rotates either clockwise or counterclockwise. The direction of this rotation with respect to the rotation direction of the first mode causes either an increase or a decrease in the number of features drawn, one off from the actual mode (Fig. S3D,E, Movies 2 and 3). As a consequence, the ‘pitch’ obtained using EFA does not correspond to actual cell features, hindering interpretation. Moreover, EFA coefficients are redundant, i.e. there are more parameters than needed to specify the same specific shape (Haines and Crampton, 2000). Consequently, comparison of cell shapes on the basis of their EFA coefficients (for example, by means of principal component analysis) is nonsensical. Together, these traits make the EFA method unsuitable for cell morphology quantification and renders meaningful comparisons between multiple cell shapes problematic.

Diaz et al. (1990) proposed a solution for the mismatch between actual shape features and EFA's results, using the fact that the relative direction of rotation is a main determinant of the reconstructed dominant harmonic or ‘pitch’ (see supplementary Materials and Methods for further details). It turns out, however, that each ellipse simultaneously contributes to two different spatial frequencies, something their heuristic solution cannot solve (Fig. S3F, Movie 4). As a consequence, although their method is often (but not always) able to recapitulate the ‘pitch’ correctly, it is never able to capture the amplitude or timbre of the cell shape correctly.

To overcome these limitations, we propose a new basis for the outline reconstruction, which we coined 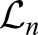, after lobe number. Similar to EFA, modes can be summed to recreate the original shape, and each mode is represented by a set of four parameters. There are also two important distinctions. First, a cell outline is now decomposed into a unique series of 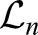 coefficients. See supplementary Materials and Methods for further details regarding elimination of coefficient redundancy. Second, shape features, such as the protrusion number (‘pitch’), their amplitude and the characteristic lobe distributions (‘timbre’), are now directly mapped to the 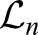 coefficients. They are obtained by decomposing each EFA harmonic into its exact, specific contributions to two separate 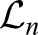 modes (Fig. 2B-D). In general, EFA modes *n*−1 and *n*+1 both partly contribute to mode 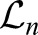, with some specific exceptions (Fig. S4). The resulting method, which we coin lobe contribution EFA or LOCO-EFA, thus consists of: eliminating multiple representations of a given outline; decomposing each *n*th EFA mode into two separate lobe contributions; and integrating those separate modes into single LOCO-EFA modes. Every 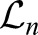 mode can be regarded as representing two oppositely rotating circles, each with its own starting point for the rotation. Each 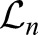 mode is composed of four coefficients corresponding to the radii and starting angles of rotation of both circles. We next assign a scalar *L_n_* value to capture the amplitude of each mode (Fig. 2D, yellow line). Quantifying the amplitude requires both the radii of and the angular distance between the starting points of the two contributing circles, as well as the starting point of the main circle, 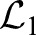, to be taken into account (see Fig. 2D and supplementary Materials and Methods for further details). The *L_n_* spectrum represents the relative contribution of each individual mode to the cell shape (Fig. S3C). Indeed, the spectrum of the six-lobed test shape used for Fig. S3C contains a pronounced peak at mode six, as well as a peak at mode one that represents the overall circular shape. To appreciate visually the contribution of specific modes, the original shape can be reconstructed using consecutive modes up to a given mode number (compare Fig. 2E with 2F).

**Fig. 3.**
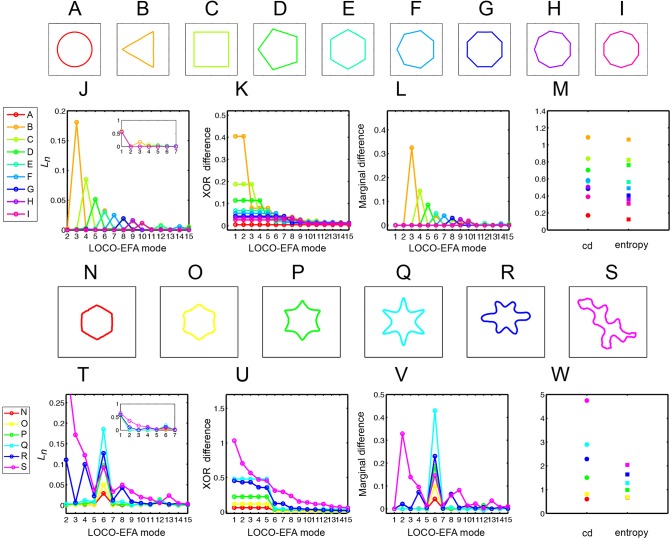
**Interpreting LOCO-EFA-derived measures for geometrical and asymmetric shapes.** (A-I) Symmetrical and well-defined geometrical shapes with normalised area. (J-L) *L_n_* (J), *XOR* (K) and marginal difference (L) profiles for the shapes shown in A-I. (J,L) For each geometric shape, a clear peak appears in the profiles, this main contributor to the shape always coinciding with the number of protrusions. (M) Cumulative difference (cd) and entropy for the shapes shown in A-I. (N-Q) Symmetrical shapes with increasing protrusion amplitude. (R,S) Asymmetrical shapes. (T-V) *L_n_* (T), *XOR* (U) and marginal difference (V) profiles for the shapes shown in N-S. Increasing protrusion amplitude leads to increasing peak levels in the profiles. Asymmetric shapes present multiple peaks, indicating that multiple modes are needed to recapitulate the original shape. (W) Cumulative difference (cd) and entropy for the shapes shown in N-S.

To illustrate how LOCO-EFA quantifies different shapes, we first apply it to geometrical shapes with variable numbers of protrusions (Fig. 3A-I). LOCO-EFA robustly determines the main LOCO-EFA mode of each shape, correctly estimating lobe number (Fig. 3J). We next tested whether LOCO-EFA also correctly captures the amplitude, by applying the method to shapes of the same ‘pitch’, but with variable amplitudes (Fig. 3N-Q). Indeed, the *L_n_* magnitude changes accordingly (Fig. 3T), its absolute value correctly measuring the size of the extensions.

Following the analogy of sound decomposition, a more nuanced quantification is timbre. Timbre resides in the entirety of the amplitude spectrum. It is determined by which overtones are emphasised in relation to one another. For cell shape studies, we consider ‘timbre’ analysis the ability to capture additional aspects of shape complexity, besides the main number and amplitude of protrusions/lobes. This additional information should enable distinction between different cellular phenotypes, such as between wild type and mutants (Lin et al., 2013). To illustrate, Fig. 3R,S shows two additional six-sided shapes that differ in ‘timbre’ from that in Fig. 3Q, with their accompanying *L_n_* spectra (Fig. 3T). For both shapes, a clear *L_6_* peak reflects their six-lobedness, and an additional peak at *L_2_*, captures the elongated nature of these shapes, and so forth. Thus, LOCO-EFA retrieves not only the main number of morphological features of a hypothetical cell, but also important fine-grained characteristics.

From the set of 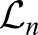 modes, additional objective metrics can be derived to help quantify different aspects of ‘cell shape complexity’. Here, we define four metrics: *XOR* difference; marginal difference; cumulative difference; and entropy.

First, cell shape complexity can be estimated from the approximation of the original shape by the first *N* LOCO-EFA modes only. It addresses how relevant each subsequent 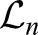 mode is for explaining that specific shape. Fig. 2F illustrated the importance of a specific mode for reconstructing the original shape (in that case, mode six). One can quantify in a straight-forward manner the relative contribution of each mode to explaining the shape by the total areal difference (either in number of grid points or μm^2^) between the original and the reconstructed shape when the first *N* LOCO-EFA modes are used. To do so, we take the *XOR* (exclusive or) between the original and reconstructed cell shapes (see Fig. 2F-H and supplementary Materials and Methods for further details). A more ‘complex’ shape requires more LOCO-EFA modes to obtain a good match. Note that a circular cell can be reconstituted using only the contribution of the first LOCO-EFA mode (*N*=1). On the other hand, cells presenting a high lobe number require a high number of modes for *XOR* to approach zero (Fig. 3K,U).

Quantifying cellular complexity can be further compressed by integrating from *n*=2 onwards the area under the *XOR* curve. We coin the resultant scalar ‘cumulative difference’ (cd), with higher values corresponding to more complex-shaped cells. Fig. 3M,W shows the cd values for the series of test shapes, indicating that cd becomes high when morphological protrusions increase in number or become larger in amplitude.

*XOR* profiles are typically not smooth. Instead, some modes peak as they strongly contribute to capturing the main shape features. Hence, the marginal decrease in the *XOR* value when an extra mode is added, coined ‘marginal difference’, further highlights the shape's dominant modes (Fig. 3L,V). This profile is comparable to the *L_n_* spectrum, also determining something akin to ‘pitch’ and ‘amplitude’. We found, however, that it bears a higher discriminatory power for more complex and irregular cell shapes. Moreover, when a cell's shape has significant contributions from multiple modes, then high marginal difference levels can be directly linked to specific cellular features (see Fig. 2G,H, Fig. 3V). Thus, marginal difference helps to identify which modes are most relevant for specific shape aspects.

Finally, shape complexity is not solely about protrusion number and amplitude, but can also arise from the irregularity of these protrusions. With the previous measures, a highly regular star-shaped cell with five outspoken lobes is quantified as being as complex as a highly distorted cell with different amplitudes and distributions of five lobes, albeit less pronounced than the star-shaped case. One might therefore prefer to define cell shape complexity as a cell's deviation from well-defined periodic outlines. A useful measure for this alternative definition of ‘cell shape complexity’ is the Shannon entropy, *E*, of the *L_n_* spectrum. The entropy measure is based upon the information content within the whole *L_n_* spectrum (Eqn 1). For many shapes, entropy yields very similar results to cumulative difference. However, they give distinct results for cell outlines that have a strong contribution from the lower modes. In such cases, entropy delivers more meaningful values regarding ‘complexity’. This is due to lower modes being able to impact cumulative difference strongly. For example, for a highly elongated cell there will be a high contribution from *L_2_*. Simply being elongated, however, does not so much represent shape complexity in the way defined above. For such a simple but elongated shape, the cumulative difference can be very similar to a shape with contributions distributed among many modes. The latter outline, however, is typically considered to be more ‘complex’. Entropy correctly captures this form of complexity. In summary, we propose LOCO-EFA and derived metrics as a new method to quantify cell shape complexity. For fully unsupervised analysis without *a priori* knowledge of cell shape features, we recommend employing all the metrics discussed in this section.

LOCO-EFA generates an infinite series of modes, without a pre-specified cut-off. Besides the measures discussed above, the *XOR* analysis also provides an algorithmic and meaningful cut-off for LOCO-EFA data analysis. Cell shapes that will be analysed with this method will in general be derived from segmentation of microscopy images. The natural choice for the grid on which to calculate the *XOR* should therefore be equivalent to the microscopy image, at its acquired resolution. *XOR* analysis (properly performed, see details in supplementary Materials and Methods) yields values that become zero when a sufficiently large, but finite, number of modes are taken into account. Additional terms then only alter the reconstructed outline at a sub-pixel resolution, i.e. at a higher resolution than the microscopy image itself. Obviously, the latter cannot be meaningful in any possible way. The mode at which *XOR* reaches zero therefore provides a natural cut-off to truncate the 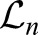 series.

### LOCO-EFA applied to plant pavement cells

To validate our method, we analysed *Arabidopsis thaliana* leaf epidermal PCs. Actual biological cells, such as PCs, can be highly asymmetrical, with multiple peaks in their *L_n_* landscape (Fig. 3S,T). The outline of an asymmetrical cell with a certain number of protrusions placed quasi-periodically along its edge results in multiple superimposed protrusion frequencies. In general, the total number of hand-counted lobes matches to a peak at the corresponding *L_n_* value (but note that hand-counting is subjective). For instance, for nine lobes a peak will be observed at *L_9_*. However, if these lobes are clustered in a pentagonal fashion, an additional peak at *L_5_* appears, and superimposed on a triangular shaped cell basis an *L_3_* contribution would be found, and so forth.

**Fig. 4.**
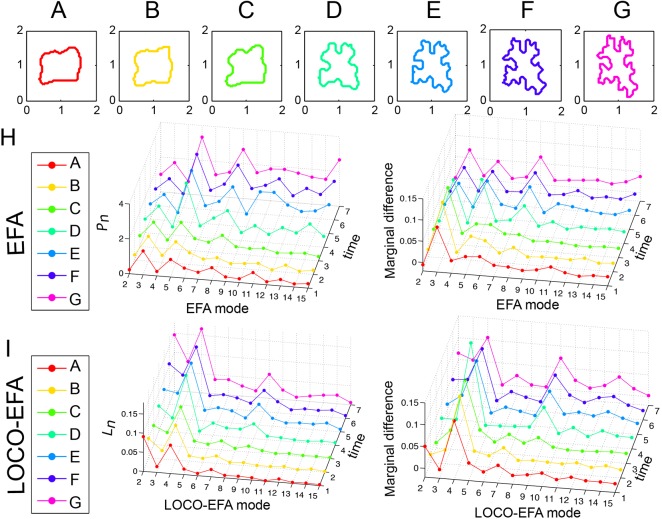
**LOCO-EFA metrics on a cell changing its shape over time.** (A-G) Sequence of a tracked PC growing over time with normalised area. (H) *P_n_* and marginal difference profiles using EFA. Applying EFA modes to approximate the cell shapes leads to erratic profiles that fail to recover the biological sequence of development, observed in the *P_n_* profile and as spurious peaks at the third and fifth harmonics in the marginal difference profile. (I) *L_n_* and marginal difference profiles using LOCO-EFA. The LOCO-EFA measurements recover the smooth transitions during the cell morphogenesis. The overall square symmetry of the cell is captured by a peak at *L_4_*, the formation of lobes by a smooth increase in *L_9_*, and later *L_13_*.

PCs acquire their characteristic jigsaw puzzle-like shape through multipolar growth patterns, such that relative simple shaped PCs become highly complex during development (Fig. 4A-G). Notably, the smooth shape changes are clearly reflected in the *L_n_* spectra over time (Fig. 4I). Its initial squarish shape and later nine- and 13-lobedness are well captured by LOCO-EFA, through peaks at modes *L_4_*, *L_9_* and *L_13_*, and corresponding peaks in the marginal difference profile. In contrast, when EFA is used, the third and fifth mode are erroneously indicated to represent shape features, besides a number of other mismatches (Fig. 4H). Importantly, the smooth cell shape development over time leads to smooth changes in the LOCO-EFA *L_n_* profile over the different time points (another example is shown in Fig. S5), in contrast to highly irregular changes in the EFA profile. This illustrates that comparably shaped cells can have very different EFA profiles, making EFA unsuitable for analysing real PC populations.

**Fig. 5.**
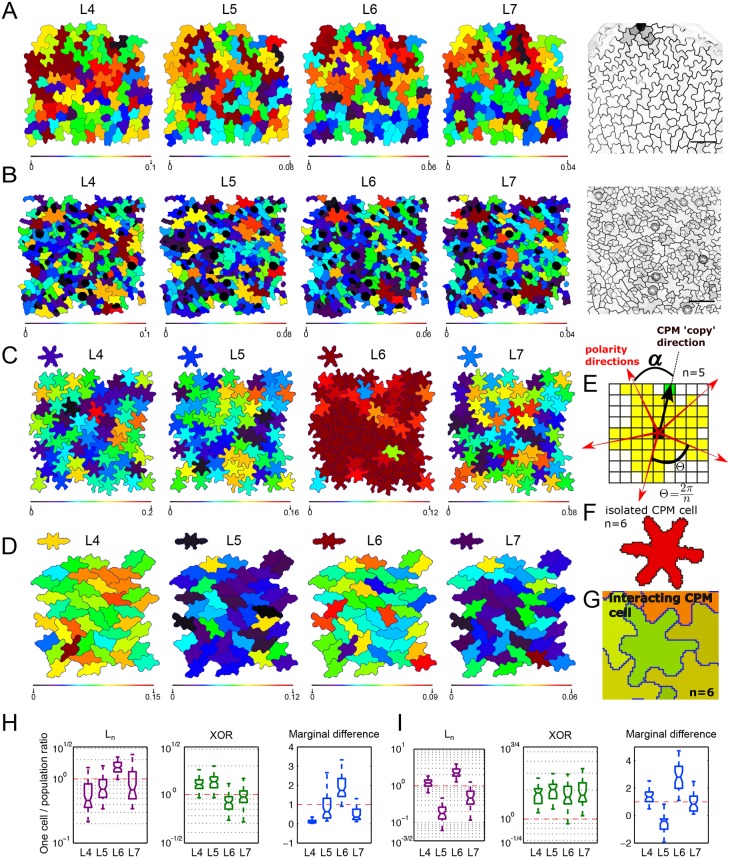
**LOCO-EFA analysis on *in vivo* and *in silico* pavement cells.** (A,B) LOCO-EFA applied to *speechless* mutant (A) and wild-type (B) leaf tissue. Colour coding depicts the *L_n_* values for four different LOCO-EFA modes, as indicated above each panel, with the scale shown below. Very few cell shapes can be reasonably captured through a single *L_n_* value, revealing cell shape complexity. (C,D) LOCO-EFA applied to *in silico* PCs reveals the degree of divergence from their specified shape that interacting cells within a tissue experience. Two different specified cell shape populations are shown (SCS1 and SCS3, each with six lobes, see Table S1). The specified shapes are depicted above each panel. Colour coding within the panels and of the specified shapes above each panel again depicts the *L_n_* values, with the scale shown below. Within the tissue, strong deviations in *L_n_* contributions are observed. (E-G) Modelling framework used to generate the *in silico* tissues. (E) Standard CPM is modified to allow for a specified number of lobes (here, *n*=5) to form at regular radial spacings (*α*). (F) This gives rise to a symmetric, multilobed specified cell shape, shown in red. (G) Within the tissue, however, cells with the same specified shape deform while interacting with neighbouring cells. (H,I) Distribution of the ratios, for three different LOCO-EFA metrics, between the cell in isolation and each of the cells within the tissue population, for SCS1 (H) and SCS3 (I), respectively. The central mark of the box plots indicates the median and the edges refer to the 25th and 75th percentiles. *n*=66 (H) and 44 (I) *in silico* cells. The red lines highlight where the ratio is unity. *L_n_* and *XOR* are plotted on a log scale.

To visualise the shape characteristics of populations of PCs, we analysed leaves of the *speechless* mutant (MacAlister et al., 2007), which does not generate during the leaf development any other cell types such as meristemoids or stomata (Fig. 1B, Fig. 5A), as well as wild-type leaf epidermis, consisting of PCs, stomata and other cells from the stomatal lineage (Fig. 1A, Fig. 5B).

Using LOCO-EFA, it is straightforward to dissect the precise contribution of each mode for each cell in the population. Fig. 5A,B shows the spatial distribution of cells within a tissue that are predominantly four-, five-, six- or seven-lobed, by colour coding cells by their *L_n_* values. Very few cells are captured by a single *L_n_* peak. Instead, the majority of shapes have significant contributions stemming from multiple modes. Consequently, simply counting the number of lobes, either manually or through automatic algorithms, would lead to incomplete information regarding the shape of such cells, making it, for example, difficult to compare mutant phenotypes. Moreover, our data shows that PCs lack a population-wide preferential *L_n_* (Fig. 5A,B).

The heterogeneity in modes that composes real populations of PCs suggests that their resultant cell shapes cannot be easily explained solely by intracellular molecular mechanisms underlying lobe and indentation patterning. Currently proposed mechanisms, based on two counteracting pathways (one for lobe formation and another for indentation formation; see details in Xu et al., 2010) give rise to Turing-like instabilities, which tend to generate symmetrical shapes (Vanag and Epstein, 2009). Moreover, these patterning models would predict that equally sized cells exhibit equal lobe numbers. However, the cell shape patterning takes place within a confluent tissue, which complicates how individual cells generate their shape. In the experimental setting, it is very hard to distinguish between the preferred shape of a cell due to its intracellular patterning, and the acquired shape due to constraints imposed by the tissue. It is well-known that if cells prefer to be round, they will take up a hexagonal shape within a tissue context (Thompson, 1917), but it is unclear what to expect for multilobed shapes. Therefore, to explore what shapes arise when a population of cells with complex shape preferences form in a confluent tissue, and to further validate LOCO-EFA on cell populations, we simulated interacting cells with pre-specified shape preferences, and employed LOCO-EFA on the resulting *in silico* tissue.

### Applying LOCO-EFA to *in silico* populations and the effect of interactions between preferred cell shapes

We create *in silico* cells using the cellular Potts model (CPM), an energy-based framework that describes cells and their dynamics through small membrane extensions and retractions (see Materials and Methods). In its basic form, CPM cell shapes emerge as a result of the interaction between interfacial tension, internal cellular pressure and cortical tension (Magno et al., 2015). Here, we used an extension of the CPM that predefines intrinsic forces causing elongation and lobedness, resulting in more complex cell shapes. This extension consists of applying additional, cell-specific forces to subcellular update events, resulting in elongated and/or multilobed preferred cell shapes (Eqn 4). Three additional forces are used that capture (1) an intrinsic tendency to elongate; (2) a tendency to form a specified number of lobes; and (3) an additional force for the cell to round up (Fig. 5E-G; J.v.R., R. Magno, V.A.G. and A.F.M.M., unpublished; Movie 5). The latter term robustly prevents cells from falling apart, which becomes important within a confluent tissue with conflicting preferred cell shapes. In the simulations, a population of cells, individually having the same preferred shape, interact with each other to form a tissue. In this way, we can compare the shape of a single cell in isolation with the shape cells attain within a tissue.

We here present the analysis for two distinct specified shapes (Fig. 5C,D; see Table S1 for the specific parameters used). Both preferred shapes have six lobes, but the cells shown in Fig. 5D also tend to be elongated. Although the same cell shape is specified for all cells within the population (above the panels, we show the acquired cell shape in isolation), local interactions within the tissue both change and diversify the cell shapes. We quantified this divergence using LOCO-EFA. For both specified shapes, the amplitudes of the main specified modes (*L_6_* in Fig. 5C and *L_2_*, *L_4_*, *L_6_* in Fig. 5D) strongly decrease within the population, whereas other modes that were not prominent in isolated *in silico* cells became relevant within the multicellular context (Fig. 5H,I). Marginal difference portrays a comparable picture, through a broadening of the set of modes involved. *XOR* analysis presents a more nuanced picture: for the elongated cells depicted in Fig. 5D a structural reduction in shape complexity is observed, i.e. the tissue context prevents cells from taking up their preferred shape complexity (Fig. 5I). For the rounded cells in Fig. 5C, however, the relative *XOR* level is smaller than that for *n*≥6, indicating additional high-mode shape complexity triggered by the cell-cell interactions. All measures indicate large cell-to-cell variations, reflecting a high shape diversity within the tissue. We further illustrate the changes in contributions and their spatial heterogeneity by colour coding *L*_*4*_-*L*_*7*_ (as indicated for each panel), for both the isolated cells and the resultant shapes of all cells within the simulated tissues. The isolated cells present a very high contribution from *L_6_*, with marginal contributions from the other modes. In contrast, owing to cellular interactions, other modes become prominent within the tissue, and vary greatly from cell to cell, even though all cells have identical specified shapes. Thus, although a cell in isolation would generate regular protrusions with specific amplitudes, periodical lobe formation becomes inhibited and gets modified within a packed tissue, with symmetry and shape distortions being directly linked to tissue packing (Fig. 5C,D,H,I). Such dynamics were observed irrespective of the specified cell shape, i.e. irrespective of the number of lobes, their amplitude, and the level of overall cell elongation, and were robust over a wide CPM parameter range (Figs S6, S7). Given that radially symmetric, periodically spaced lobed cell shapes are highly unlikely to be space filling, resolving conflict between preferred shape and confluency could be a relevant driving force for complex cell shape morphogenesis.

### LOCO-EFA applied to *Drosophila* amnioserosa cells

**Fig. 6.**
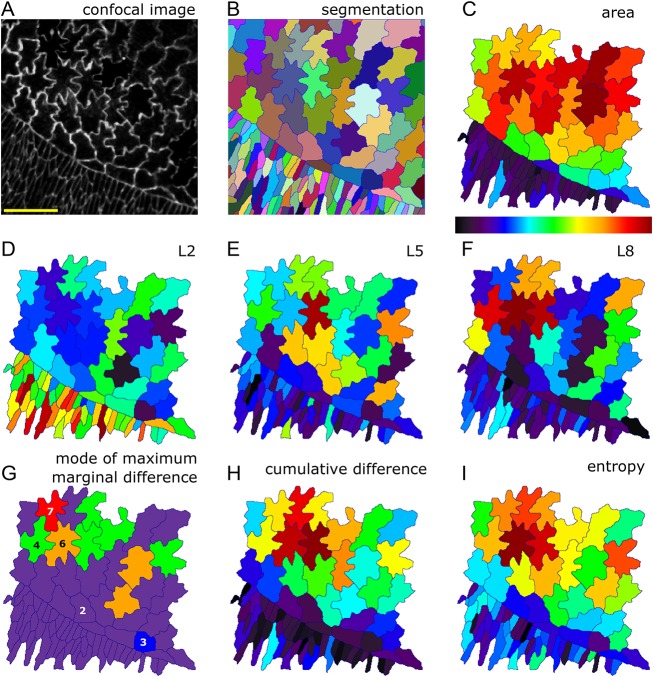
**Cell shape analysis during dorsal closure of the *Drosophila* embryo.** (A) Confocal image of amnioserosa cells. (B) Segmentation identifies each cellular domain by a unique ID, represented by a distinct colour. (C-I) Several cell shape characteristics, quantified and depicted by the heat map shown below C. (C) Cell area. Amnioserosa cells are larger (red) than surrounding epithelia (blue and purple cells). (D) *L_2_* for each cell. Levels are high in surrounding epithelia, corresponding to predominant cell elongation. (E) *L_5_* for each cell. Amnioserosa cell shapes carry larger representations of higher mode numbers. The cell with the highest *L_5_* contribution does indeed display five distinct protrusions. (F) *L_8_* for each cell. Higher modes substantially contribute to the amnioserosa cells, with *L_8_* strikingly high for the cell with eight visibly prominent protrusions, and high for other multilobed shapes. (G) Mode at which the highest marginal difference occurs, depicted for each cell. Colours represent mode numbers, as indicated. For example, many cells can be described as having a predominantly elongated axis (purple cells, with highest mode 2), whereas one cell is best described as being triangular (blue cell, with highest mode 3), etc. (H) Cumulative difference for each cell, a measure of lobe richness (both number and amplitude). (I) Entropy for each cell, a measure of shape irregularity. Colour scale is between 0 and maximum for D-F and between minimum and maximum for C,H,I.

To demonstrate LOCO-EFA's applicability to other (non-plant) developing tissues in which cells present a high level of shape complexity, we analysed *Drosophila* during dorsal closure (Knust, 1997). Amnioserosa, the squamous epithelial that covers the dorsal side of the embryo, undergoes dramatic cell shape changes during this morphodynamic event. Simple cuboidal to columnar epithelium covers the remainder of the embryo, with both captured in our image (Fig. 6A,B). At the imaged interface, cells present a broad distribution in size (Fig. 6C) and shape complexity. Analysing the spatial distribution in the magnitude of the different LOCO-EFA modes (Fig. 6D-F) reveals how the surrounding epidermis can be described by cell elongation alone (very high and dominant *L_2_* values), whereas the amnioserosa cells are characterised by higher LOCO-EFA modes (with Fig. 6E and 6F showing *L_5_* and *L_8_*, respectively). Differences in their patterning represent cell-to-cell variations in lobe numbers. These shape characteristics are consistent with classical studies (Young et al., 1993), which proposed that observed elongation of epidermal cells perpendicular to the long axis of the embryo could explain the change in surface area required to cover the amnioserosa. The cell shapes can be analysed further by depicting the mode corresponding to the maximum marginal difference for each cell. This indicates the dominant number of extensions best describing that shape, ‘counting’ their major morphological feature (Fig. 6G), and shows how elongation dominates in the epidermis whereas higher modes dominate in the amnioserosa. The cumulative difference is a measure of lobe richness, its value increasing as number and amplitude of lobes increases. The cumulative difference yields highest values for the multilobed cells within the amnioserosa, and presents low levels for the epidermal cells (Fig. 6H). Entropy provides an alternative quantification of shape complexity, by measuring shape irregularity. Highly asymmetric cells require a broad range of modes to capture their shape, leading to high entropy values. The spatial distribution of entropy (Fig. 6I) is similar to the spatial distribution of cumulative difference, with differences between the two being particularly interesting, entropy directly highlighting the most irregular cells. In short, LOCO-EFA and its derived quantifications retrieve both the level and type of shape complexity of both *Arabidopsis* PCs and *Drosophila* amnioserosa.

## DISCUSSION

Recent progress in microscopy and imaging techniques generates a need for adequate analytical tools to capture relevant information efficiently and objectively (Zhong et al., 2012). Image acquisition through high-throughput microscopy generates large datasets beyond the human ability (or patience) to be analysed manually, demanding computational tools. We have developed a new analytical tool that takes as the input the contour of a two-dimensional cell projection, extracting from it, in an efficient and parameter-independent manner, quantitative meaningful shape information. Importantly, the pipeline can be integrated within segmentation procedures (Fernandez et al., 2010; J.v.R., J. A. Fozard, R. Carter, M.H., Y.E.S.-C., R. Sablowski, V.A.G. and A.F.M.M., unpublished), to fully automate shape analysis of a series of images.

Our method can be intuitively grasped through the analogy of music perception. To quantify an instrument playing a certain note, say a violin playing the note A, one first needs to have a device that determines the note played. We have shown here that LOCO-EFA, unlike EFA, correctly determines the analogous feature for shapes, which is the number of protrusions. Moreover, LOCO-EFA, in contrast to EFA, quantitatively measures the amplitude of that particular feature; this is similar to determining the volume of a given note, when multiple notes are played concomitantly. In all examples presented here we have normalised to cell area. Hence an L-value of 0.15 indicates a peak-to-trough distance of 15% of the cell diameter (amplitude equal to 15% of the cell radius).

LobeFinder, the recent method developed by Wu et al. (2016) can also be employed to assess protrusion number. When the biological question asked requires not only the ‘pitch’ to be measured, but also the ‘volume’ and ‘timbre’, corresponding to lobe amplitude and other irregularities, such alternative methods are insufficient. Indeed, LOCO-EFA provides a holistic set of measurements that allows complex morphologies to be quantified in a reproducible manner.

We illustrate how the measurements obtained via LOCO-EFA can be interpreted, first using simple shapes (geometrical or symmetrical forms), followed by using confocal images of *Arabidopsis* PCs and *Drosophila* amnioserosa, to assess the performance of our method on actual, highly complex and asymmetric biological shapes. When analysing complex shapes through the *L_n_* spectrum only, it is non-trivial to ‘visualise’ the corresponding shape in the same manner as can be done for geometric shapes. In such cases, it is useful to plot the *XOR* and marginal difference profiles, to gain a better notion of the major shape properties. PC shape analysis is directly biologically relevant, because many of the players accounting for the lobe and indentation patterning are known (Jones et al., 2002; Xu et al., 2010), enabling one to extend the study of cell shape control to mutants and experimental interferences. We found that few cells have a symmetrical shape, i.e. most cannot be represented well by a single high *L_n_* value. It is unlikely that such composition of real cell shapes in several *L_n_* values can be fully explained by the existence of two counteracting pathways specifying lobe and indentation identity. Our *in silico* approach rather suggests that the interactions between space-filling shapes can dramatically increase the overall irregularity: even when the CPM cells are specifically programmed to develop well-defined regular shapes, the interactions between them trigger dramatic cell shape deviations and variations. Within the tissue the main, specified mode decreases in strength and the other modes become relevant. Thus, tissue confluency leads to asymmetric and variable resultant shapes.

Although our synthetic data is but a phenomenological description of real shapes, our results suggest that the local influence of neighbours during PC development could be important for shape acquisition. To assess this hypothesis further, it will be crucial to perform quantitative shape analysis on *in vivo* cell populations over time, combined with growth tensor analysis (i.e. anisotropy and spatial patterning in the growth rate). Such studies, combined with genetic or physical perturbations in cell growth and deformation and *in silico* cell growth models, could help untangle how cell shape specified at the cellular level is linked to the resultant shape arising at the tissue level.

Applying LOCO-EFA to cell-tracking data, we observed that the LOCO-EFA profiles of those changing cells varied smoothly over time. Such trajectories are cell specific and provide unique fingerprints of each individual developing cell. This opens the possibility of using the *L_n_* spectrum as cell identifiers within a temporal sequence of images, to help track populations of cells automatically.

To illustrate how this powerful tool can be used to measure complex undulating cells, we have here applied LOCO-EFA to *Arabidopsis* PCs and *Drosophila* amnioserosa. Although we focussed on discussing overall shape distributions throughout the tissue, LOCO-EFA shape descriptors could also be used to investigate correlations in shape between neighbouring cells, in a similar manner to investigations of topological traits in the same tissue (Carter et al., 2017). Moreover, LOCO-EFA analysis on shape dynamics and shape correlations between neighbouring cells can be easily extended to other cell types and other species, including less complex shapes. Furthermore, LOCO-EFA could also be relevant for understanding phenotypic morphology of subcellular structures, such as mitochondria, which can present different levels of shape complexity (Dimmer et al., 2002), and sperm cell nuclei, which have already been analysed using EFA (Mashiko et al., 2017). Our method is also well-suited for studying organ shape development, specifically when landmarks are difficult to assign. It could, therefore, be used to improve quantification and biological meaningfulness of previous EFA-based studies that, for example, decomposed entire leaf shapes (Liao et al., 2017), insect wings (Yang et al., 2015), jaw shape and sizes (Rose et al., 2015) and pinniped whisker morphologies (Ginter et al., 2012). LOCO-EFA can even be employed at different levels within the same organism, for example to quantify leaf shape and serrations as well as root morphology (Li et al., 2017 preprint). Lastly, LOCO-EFA could constitute a powerful tool for whole organism analysis, especially within paleobiology, where it could enrich current elegant studies initiated using EFA, to, for example, analyse bivalves (Crampton, 1995), trilobite-like arthropod evolution (Jackson and Budd, 2017) and Triatominae eggs (Santillán-Guayasamín et al., 2017). For all such studies, when possible, we recommend that our method be integrated with recent image analysis pipelines, allowing extraction and analysis of shape information in a high-throughput manner (Heller et al., 2016; Stegmaier et al., 2016).

In short, LOCO-EFA can be used to quantify morphologies described as closed two-dimensional contours, across scales, from the subcellular level to organs and beyond.

## MATERIALS AND METHODS

### Confocal images and image processing

Columbia wild-type or *speechless* mutant (MacAlister et al., 2007) leaves expressing pmCherry-Aquaporin (Nelson et al., 2007) were imaged using a confocal microscope Leica SP5 at comparable stages and in comparable regions. Cells changing over time were imaged using a custom-made perfusion chamber (Kuchen et al., 2012; Robinson et al., 2011; Sauret-Güeto et al., 2012). Further image processing to flatten the images was performed using ImageJ. *Drosophila melanogaster* embryos expressing ubi-DE-Cadherin-GFP (Oda and Tsukita, 2001) were dechorionated in bleach, rinsed in water and attached to a coverslip with the dorsal side up using heptane glue and covered with Halocarbon Oil 27 for live imaging on a Zeiss 780 confocal. Both the *Arabidopsis* and *Drosophila* images were segmented using in-house software (segmentation Potts model; J.v.R., J. A. Fozard, R. Carter, M.H., Y.E.S.-C., R. Sablowski, V.A.G. and A.F.M.M., unpublished). In this study, we present a single, typical example of a wild-type and of a *spch* leaf, as well as five typical examples of static PC outlines and two typical examples of developing PCs, all within a *spch* leaf. These images were selected from a study in which one wild-type and seven *spch* leaves were imaged at in total 15 time points for the wild-type leaf and 121 time points for the *spch* leaf (Carter et al., 2017). The amnioserosa image represents a typical example selected from four live-imaged embryos.

### Shape descriptors

Average lobe lengths and neck widths were calculated using ImageJ (Analyse→Measure). The skeleton was calculated using ‘Better Skeletonization’ by Nicholas Howe, available through MATLAB File Exchange (https://uk.mathworks.com/matlabcentral/fileexchange/11123-better-skeletonization?focused=5073847&tab=function).

### Geometric shapes

All geometric shapes were generated by the ‘superformula’ described by Gielis (2003), and were analysed in the same manner as the confocal images.

### XOR

All the grid points belonging to each individual real or synthetic PC were compared with all the grid points captured by the subsequent series of LOCO-EFA reconstructions. A reconstruction of level *N* takes into account the first *N* 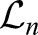 modes. The *in silico* cells were generated using the cellular Potts model, which is a grid-based formalism, whereas for the experimental data the grid points were directly defined by the imaging resolution. The scripts used to calculate the *XOR* and to colour code the real and synthetic cells were written in the coding language C. See supplementary Materials and Methods for further details.

### Entropy and other measurements

The entropy measure is defined as:
(1)
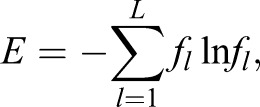
where *f*_*l*_ refers to the relative proportion of each *L_l_* for a given *L* number of modes analysed, i.e. 
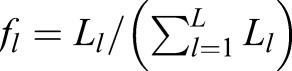
.

Shape approximations, cumulative difference and entropy were calculated using the first 50 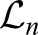 modes. To capture cell shape complexity linked to protrusions rather than mere anisotropy, cumulative difference is calculated from the second 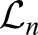 mode onwards. This value turned out to be more than sufficient to capture any cell shape given the grid point resolution used for all cases here. Note, however, that very high-resolution images might require additional modes to fully capture the shape.

### Cellular Potts model generating complex cell shapes

The cellular Potts model (CPM) is an energy-based model formalism used to model cellular dynamics in terms of cell surface mechanics (Magno et al., 2015). Individual cells are described by a set of grid points on a lattice. In this article, we used the CPM to generate *in silico* cells with relatively complex shape preferences that are allowed to interact within a confluent setting. During each simulation step, a grid point is chosen in a random fashion to evaluate whether its state changes into one of its neighbouring states, effectively corresponding to a small cell shape modification at that point. To evaluate whether such state change will occur, the energy change is calculated that such a copy would cause. This is done by calculating the change in the configurational energy as defined by the following Hamiltonian, which sums up the energy contribution of each pixel within the entire field as well as of all cells:
(2)
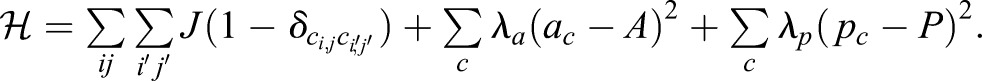
*J* refers to the coupling energy, summed over all grid points (*i*, *j*) and their eight (second order) neighbours (*i*′, *j*′). The Kronecker delta term 
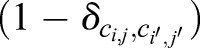
 simply assures that neighbouring lattice sites of the same state (i.e. belonging to the same cell) do not contribute to the total energy of the system. The variables *a_c_* and *p_c_* denote, respectively, the actual cell area and the actual cell perimeter for each cell (*c*); the parameters *A* and *P* denote the target cell area and perimeter. The parameters *λ_a_* and *λ_p_* describe the resistance to deviation from the target area and perimeter, respectively. The probability a copying event is accepted depends on the change in the Hamiltonian, 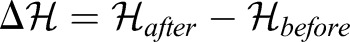, in the following way:
(3)
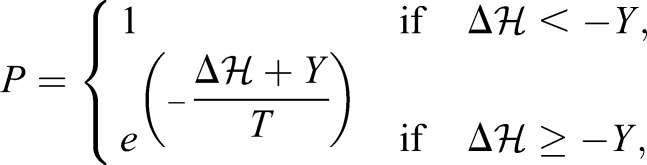
where *Y* corresponds to the yield or ability of a membrane to resist a force and *T* (simulation temperature) captures additional stochastic fluctuations. Copying events that decrease 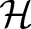 by at least *Y* will always be accepted, otherwise acceptance follows a Boltzmann probability distribution (Eqn 3).

To generate cells with a particular number of preferred protrusions, we modify the change in the Hamiltonian as calculated for every evaluated copying event, effectively shortcutting intracellular biochemistry and biophysics, in the following way. Simulated cells are attributed with a specified preferred number of lobes, amplitude of lobes, overall elongation and roundness, implemented by modifying the change in the Hamiltonian for every evaluated copy event as follows (J.v.R., R. Magno, V.A.G. and A.F.M.M., unpublished):
(4)
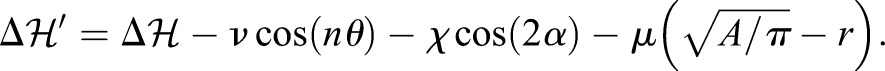
Those three additional terms are evaluated for both cells involved in the copying event, so there are effectively six additional terms. The first term captures the tendency to form *n* lobes, with *ν* capturing the propensity to extend to form a lobe or to retract to form an indentation, thus giving rise to the amplitude or pointedness of the lobes. *θ* describes the angle between any of the *n* equally spread out target directions for outgrowth and the vector determined by the coordinates of the grid point under evaluation and the centre of mass of the cell (hereafter called the copy vector) (Fig. 5E). To clarify, when a cell extension is considered right on top of one of the target directions, then *nθ*=0, cos(*nθ*)=1, and tendency to extend is maximally increased, whereas halfway between two target directions, *nθ*=π, cos(*nθ*)=−1, and the tendency to extend is maximally suppressed.

The second term in Eqn 4 captures an overall elongation, implemented in a similar fashion. The parameter *χ* corresponds to the propensity to elongate and *α* is the angle between the elongation vector and the copy vector.

If only these two terms are used, cells within tissue simulations can easily lose coherence, i.e. fall apart. Therefore, a third term was added, capturing a propensity to roundness. The parameter *μ* captures the resistance of a cell to deviate from a circle, with *r* being the length of the copy vector, and 
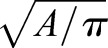
 being the preferred radius of cell, given its target area.

Importantly, the target lobe and elongation vectors are not fixed during the simulation. At intervals of 100 simulation time steps they are dynamically updated, in order to attain the most favourable position, effectively ‘accommodating’ its lobe positions with respect to its neighbours. During a vector update step, the preferred directions of extension are matched to the set of directions for which the current shape of cells presents the strongest level of extension.

The initial cell positions within the field were randomly chosen. Simulations were run for 10,000 time steps (see an example in Movie 5. Parameters used for each used specified cell shape are given in Table S1.

## Supplementary Material

Supplementary information

Supplementary information
