# Supplementary material for: Morphometrics of complex cell shapes: lobe contribution elliptic Fourier analysis (LOCO-EFA)
Source: Development. 2018 Mar 15;145(6):dev156778. doi: 10.1242/dev.156778 (PMC5897594; doi:10.1242/dev.156778)
Supplement: Supplementary information [file develop-145-156778-s1.pdf]

# Supplementary Materials and Methods

## Decomposing shape: Lobe Contribution Elliptical Fourier Analysis (LOCO-EFA)

In this section, we first summarise previous efforts to make EFA coefficients interpretable within a morphometrics perspective and explain why matching EFA coefficients with shape features generally does not hold. We describe in detail our new method, Lobe Contribution Elliptical Fourier Analysis (LOCO-EFA). We show how it provides quantitative and biologically interpretable measurements that are unique for a given shape, overcoming the shortfalls of the previous methods.

### Standard Fourier Analysis cannot be used to quantify complex cell shapes

Standard Fourier Analysis has been widely used to analyse cell morphology. It can, however, only be applied when cells present simple holomorphic shapes, i.e., when the radii emanating from the centroid of a cell intersect its outline only once (Figure S2A and Pincus and Theriot (2007)). When the geometry of a cell is more complex, as in the case of pavement cells, and radii emanating from the centroid can intersect the outline more than once, the shape cannot be decomposed using a Fourier expansion based on polar coordinates (Figure S2B and Schmittbuhl et al. (2003)).

### Elliptical Fourier Analysis fails to align mode frequency with morphological features

In 1982 Kuhl and Giardina proposed the Elliptical Fourier Analysis (EFA) to describe the contour of any two-dimensional shape (both holomorphic and non-holomorphic), derived from the coordinates of all the points along its outline.

In short, EFA takes the $x$ and $y$ coordinates of a closed contour and decomposes it into an infinite summation of related ellipses:

$$x(t) = \alpha_0 + \sum_{n=1}^{\infty} \left( \alpha_n \cos\left(\frac{2n\pi t}{T}\right) + \beta_n \sin\left(\frac{2n\pi t}{T}\right) \right), \tag{5a}$$

$$y(t) = \gamma_0 + \sum_{n=1}^{\infty} \left( \gamma_n \cos\left(\frac{2n\pi t}{T}\right) + \delta_n \sin\left(\frac{2n\pi t}{T}\right) \right), \tag{5b}$$

where $\alpha_n$, $\beta_n$, $\gamma_n$ and $\delta_n$ are the so-called EFA coefficients and $\alpha_0$ and $\gamma_0$ are the $x$- and $y$-offset of the initial contour. The detailed derivation of the formulae for $\alpha_0$, $\gamma_0$, $\alpha_n$, $\beta_n$, $\gamma_n$ and $\delta_n$ can be found in Kuhl and Giardina (1982). They are calculated from a discrete chain of contour points $(x_i, y_i)$ with $i = 1, \ldots, K$ (see Figure S3A), $K$ being the total number of points along the closed contour. We define $(x_0, y_0) \equiv (x_K, y_K)$, given that cell contours are closed. Now imagine drawing the contour of the cell, then $\triangle t_i$ is the time spent drawing the line segment of the contour that links $(x_{i-1}, y_{i-1})$ to $(x_i, y_i)$, i.e., $\triangle t_i = \sqrt{(x_i - x_{i-1})^2 + (y_i - y_{i-1})^2} = \sqrt{\triangle x_i^2 + \triangle y_i^2}$. Note that $\triangle t_i$ is not fixed but can vary for each interval. Define $T$ as the total time spent to draw the whole contour, i.e., $T = \sum_{i=1}^{K} \triangle t_i$. The "time" passed while drawing the contour, starting from contour point $(x_0, y_0)$, or, equivalently, the distance passed along the contour to reach each contour



Development • Supplementary information

point $(x_i, y_i)$, is referred to as $t_i$, i.e., $t_i = \sum_{p=1}^{i} \triangle t_p$, with $t_0 = 0$, and $t_K = T$, the "total drawing time" or total perimeter length (see Figure 1 in the main text). Given that no equal spacing between the points is required, it is straightforward to define $K$ observation points from any kind of cell contour. The only requirements are that the contour is closed and the coordinates form an ordered list that follows the contour. The EFA coefficients are then given by:

$$\alpha_n = \frac{T}{2n^2\pi^2} \sum_{i=1}^{K} \frac{\triangle x_i}{\triangle t_i} \left( \cos \frac{2n\pi t_i}{T} - \cos \frac{2n\pi t_{i-1}}{T} \right) , \tag{6a}$$

$$\beta_n = \frac{T}{2n^2\pi^2} \sum_{i=1}^{K} \frac{\triangle x_i}{\triangle t_i} \left( \sin \frac{2n\pi t_i}{T} - \sin \frac{2n\pi t_{i-1}}{T} \right) , \tag{6b}$$

$$\gamma_n = \frac{T}{2n^2\pi^2} \sum_{i=1}^{K} \frac{\triangle y_i}{\triangle t_i} \left( \cos \frac{2n\pi t_i}{T} - \cos \frac{2n\pi t_{i-1}}{T} \right) , \tag{6c}$$

$$\delta_n = \frac{T}{2n^2\pi^2} \sum_{i=1}^{K} \frac{\triangle y_i}{\triangle t_i} \left( \sin \frac{2n\pi t_i}{T} - \sin \frac{2n\pi t_{i-1}}{T} \right) . \tag{6d}$$

The offset to the contour is given by:

$$\alpha_0 = \frac{1}{T} \sum_{i=1}^{K} \left( \frac{\triangle x_i}{2\triangle t_i} \left(t_i^2 - t_{i-1}^2\right) + \xi_i (t_i - t_{i-1}) \right) + x_0 , \tag{7a}$$

$$\gamma_0 = \frac{1}{T} \sum_{i=1}^{K} \left( \frac{\triangle y_i}{2\triangle t_i} \left(t_i^2 - t_{i-1}^2\right) + \varepsilon_i (t_i - t_{i-1}) \right) + y_0 . \tag{7b}$$

where $\xi_i = \sum_{j=1}^{i-1} \triangle x_j - \frac{\triangle x_i}{\triangle t_i} \sum_{j=1}^{i-1} \triangle t_j$, $\varepsilon_i = \sum_{j=1}^{i-1} \triangle y_j - \frac{\triangle y_i}{\triangle t_i} \sum_{j=1}^{i-1} \triangle t_j$, and $\xi_1 = \varepsilon_1 = 0$. Further details and full derivation can be found in Kuhl and Giardina (1982).

Each set of four coefficients yields an ellipse (also referred to as the "$n$th mode" or "$n$th elliptic harmonic"), with a certain orientation and a certain starting point. The original cell outline can thus be expressed as an infinite summation of ellipses. Note that $x(t)$ and $y(t)$ are periodic functions with period equal to $T$.

A visual way to understand how the set of ellipses gives rise to the final shape is as follows: the second elliptic harmonic traces two clockwise or counter-clockwise revolutions around the first harmonic; the third harmonic traces three revolutions around the path drawn by the second harmonic; and the $n$th harmonic traces $n$ revolutions around the path drawn by the previous harmonic (see Figure S3 and Movie 1).

Diaz et al. (1990) proposed a heuristic measure regarding the contribution of each harmonic to the shape through an approximation of the perimeter of each ellipse multiplied by its harmonic number $n$:

$$P_n = 2\pi n \sqrt{\frac{\lambda_{1n}^2 + \lambda_{2n}^2}{2}} , \tag{8}$$

where $\lambda_{1n}$ and $\lambda_{2n}$ are the major and minor axis of the $n$th ellipse. Moreover, Diaz et al. (1990) introduced an additional correction to capture the complex relationship between EFA modes and shape feature periodicity.



Development • Supplementary information

The direction of rotation of the $n$th harmonic ellipse is given by the determinant of the EFA coefficients matrix, $\det \begin{bmatrix} \alpha_n & \beta_n \\ \gamma_n & \delta_n \end{bmatrix}$, i.e., the direction of rotation is given by the sign of

$$r_n = \alpha_n \delta_n - \beta_n \gamma_n \,. \tag{9}$$

If $r_n < 0$, the elliptic harmonic is rotating clockwise; if $r_n > 0$ the elliptic harmonic is rotating counter-clockwise. When EFA is used for shape approximation, mode $n$ contributes to shape features with an $n+1$ or $n-1$ periodicity. This is in contrast to standard Fourier Analysis, in which mode $n$ contributes to shape features with an $n$ periodicity. Standard Fourier Analysis, however, is only possible for holomorphic shapes, and hence cannot be applied to, for example, pavement cells. Diaz et al. (1990) observed that whether mode $n$ predominantly contributes to shape features with an $n+1$ or with an $n-1$ periodicity strongly depends on whether the $n$th harmonic rotates together with or against the direction of the first harmonic (see Movie 2 and Movie 3). This effect of presenting contributions to the $n+1$'th and $n-1$'th mode depending on the rotation direction of the first and $n$th harmonic is a common phenomenon observed for objects orbiting around others (hereafter referred to as the relative direction effect). A well-known example of the relative direction effect is the rotation of the Earth and its movement around the sun. The actual number of rotations our planet makes per year (as observed from "star-rise to star-rise", the so-called sidereal days) is one off from the number of days we perceive in a year (from "sunrise to sunrise", the so-called solar days). Because our planet rotates around its axis in the same direction as it moves around the Sun, the number of solar days per year is 365, one less than the number of sidereal days per year, which is 366. If the rotation of Earth would have been in the opposite direction as its movement around the sun, the number of solar days per year would instead have been 367. In light of exactly the same principle, Diaz et al. (1990) introduced that when the $n$th elliptic harmonic is moving in the same direction as the first harmonic, its shape contribution $P_n$ should be assigned to $n-1$; inversely, when the direction of a given mode is opposite to the first harmonic, its shape contribution $P_n$ should be assigned to $n+1$.

We will show below that this simple heuristic is reasonable as long as the ellipse marginally deviates from a circle, but is is not valid in general. When the aspect ratio of the ellipse ($\lambda_{1n}/\lambda_{2n}$) is large (i.e., the elliptical harmonic is very flat, deviating significantly from a circular shape), the proposed rule fails to apply. Figure S3F illustrates a situation when the rotation direction of the first and third harmonic are opposite (and no other modes are used), yet instead of generating a contour with $n-1=2$ protrusions, as expected from the heuristic rule, a four-sided outline is generated, clearly illustrating that this method of $P_n$ shifting does not work in general (see also Movie 4). Moreover, it is not possible to reconstruct the original shape using the $P_n$ values, and therefore cannot be used for additional analysis based on shape reconstruction as presented in the main paper. This strongly limits usage of EFA for biological shape interpretations and statistical population analysis. Surprisingly, although EFA has been used to quantify morphology at the organ level, relative direction effect has typically been ignored altogether (Chitwood et al., 2013; Frieß and Baylac, 2003; Iwata et al., 2010, 1998; Neto et al., 2006; Yoshioka et al., 2005).

Realising that the source of the problem is linked to the eccentricity of the ellipses, it became clear to us that we could overcome this issue by essentially decomposing each ellipse into two counter-rotating circles. All circles can then be redistributed, forming a

new base. The details of how to do so are discussed below. We call the new base $\mathcal{L}_n$, which, when summed up, can also reconstitute the original shape.

## Contouring the limitations: Lobe Contribution Elliptical Fourier Analysis (LOCO-EFA)

To capture the biologically relevant cell shape features, overcoming the limitations of using $P_n$ and rotation-dependent $n+1$, $n-1$ adjustments, we have developed an alternative method coined Lobe Contribution Elliptical Fourier Analysis (LOCO-EFA). As the name indicates, it correctly maps the contribution of each mode/harmonic to the corresponding morphological features. This is done by separating each elliptic harmonic into two circular harmonics, each rotating in an opposite direction.

First we rewrite the EFA (Equation 5) in matrix form:

$$\begin{bmatrix} x(t) \\ y(t) \end{bmatrix} = \begin{bmatrix} \alpha_0 \\ \gamma_0 \end{bmatrix} + \sum_{n=1}^{N} \begin{bmatrix} \alpha_n & \beta_n \\ \gamma_n & \delta_n \end{bmatrix} \begin{bmatrix} \cos\left(\frac{2n\pi t}{T}\right) \\ \sin\left(\frac{2n\pi t}{T}\right) \end{bmatrix} , \tag{10}$$

with the infinite sum being truncated at the $N$th order harmonic.

Equation 10 can concisely be expressed as

$$[X(t)] = [A_0] + \sum_{n=1}^{N} [A_n] [M_n(t)] , \tag{11}$$

in which $[X(t)]$ corresponds to the drawn cell outline $\begin{bmatrix} x(t) \\ y(t) \end{bmatrix}$; $[A_0]$ represents the spatial offset $\begin{bmatrix} \alpha_0 \\ \gamma_0 \end{bmatrix}$; $[A_n]$ corresponds to the EFA coefficients matrix $\begin{bmatrix} \alpha_n & \beta_n \\ \gamma_n & \delta_n \end{bmatrix}$; and $[M_n(t)]$ refers to the rotor $\begin{bmatrix} \cos\left(\frac{2n\pi t}{T}\right) \\ \sin\left(\frac{2n\pi t}{T}\right) \end{bmatrix}$. (For clarity, we will use the notation [..] throughout to emphasise we are dealing with matrices; not to be confused with |..| that represents determinant, which we here only refer to as det [..].)

The LOCO-EFA method consists of three steps: 1) eliminate multiple representations of the same outline; 2) decompose each *nth* elliptic harmonic into two circular harmonics, each rotating in an opposite direction; and 3) determine $\mathcal{L}_n$ and $L_n$ for all $N$ modes. Below we describe these steps in detail.

### (1) Eliminate multiple representations of the same outline

It had already been noted that EFA coefficients are redundant and therefore compromise statistical analysis and shape comparisons (Haines and Crampton, 2000). We found that there are three sources of degeneracy in the EFA coefficients that therefore have to be eliminated. First, a contour can be drawn starting from any arbitrary initial point along the contour. While exactly the same outline is drawn, each starting point is represented by a completely different set of EFA coefficients for all modes $[A_n]$. Basically, whenever the starting point is changed, all elliptic harmonics take a different orientation (Kuhl and Giardina, 1982). The first step is therefore to transform the EFA coefficients such that the starting point of the first harmonic is always positioned at, for standardisation, the extreme of the semi-major axis (see further below). The second source of degeneracy, however, is that such a normalisation still allows for two possible representations of the



Development • Supplementary information

outline, since each of the two extremes along the semi-major axis can be chosen as the starting point. Moreover, a third source of degeneracy is due to the fact that the outline can be drawn clockwise or counter-clockwise. Clearly, all three sources of degeneracy have to be removed to make any comparison between cells sensible.

The first step is to determine where the new starting point should be positioned, as well as the scaled amount of time or temporal angle $\left(\tau_1 = \frac{2\pi t_1}{T}\right)$ required to reach the starting point (see Kuhl and Giardina, 1982). As stated above, we wish the starting point to coincide with one of the extremes of the semi-major axis of the first harmonic. Points along the first harmonic $(x_1, y_1)$ can be described as :

$$x_1(\tau) = \alpha_1 \cos\tau + \beta_1 \sin\tau \,, \tag{12a}$$

$$y_1(\tau) = \gamma_1 \cos\tau + \delta_1 \sin\tau \,, \tag{12b}$$

with $\tau = \frac{2\pi t}{T}$ being the scaled time or temporal angle. By differentiating the magnitude of the first harmonic ellipse $E(\tau)=\sqrt{x_1(\tau)^2 + y_1(\tau)^2}$ and setting its derivative to zero $\left(\frac{dE(\tau)}{d\tau} = 0\right)$, the temporal angles can be found at which the extremes along the semi-major and semi-minor axes of the first harmonic are reached (Kuhl and Giardina, 1982):

$$\tau_1(\nu) = \frac{1}{2}\arctan\left(\frac{2(\alpha_1\beta_1 + \gamma_1\delta_1)}{\alpha_1^2 + \gamma_1^2 - \beta_1^2 - \delta_1^2}\right) + \frac{\nu}{2}\pi \,. \tag{13}$$

The values $\nu = 0, 1, 2, 3$ give the four possible solutions along both the axes, after which the same points get repeated. For LOCO-EFA, it is required (see further below) to limit the starting point to the semi-major axis only. To satisfy this condition, the second derivative of $E(\tau)$, evaluated at the temporal angle, should be negative, i.e., $\left.\frac{d^2E(\tau)}{d\tau^2}\right|_{\tau_1} < 0$. Substituting the found solutions into the second derivative results in $\nu = 0$ and $\nu = 2$ belonging to the points along the semi-major axis whenever the denominator of the arctan term is positive, and the solutions $\nu = 1$ and $\nu = 3$ belonging to the points along the semi-major axis whenever the denominator of the arctan term is negative. A very straightforward computational implementation of this result is to make use of the four-quadrant inverse tangent function (atan2) as provided by most programming languages (i.e., such that $\mathrm{atan2}(1, 1) = \pi/4$ is different from $\mathrm{atan2}(-1, -1) = -3\pi/4$). Then, using

$$\tau_1 = 0.5\mathrm{atan2}\left(2(\alpha_1\beta_1 + \gamma_1\delta_1), \alpha_1^2 + \gamma_1^2 - \beta_1^2 - \delta_1^2\right) \tag{14}$$

automatically and unambiguously ensures that the temporal angle $\tau_1$ is located at one of the extremes of the semi-major axis.

This still leaves two ways to position the starting point (one for each of the extremes of the semi-major axis) and thereby two distinct representations of a same outline. We therefore further restrict $\tau_1$ to always lie within the first or second quadrant (I or II in Figure S8A). This is achieved by testing if the obtained $\tau_1$ that shifts the starting point of the first harmonic to the semi-major axis indeed positions it within quadrant I or II. To shift the starting point, we first introduce a time shift $\tau' = \tau - \tau_1$ such that at $\tau' = 0$ the first harmonic is positioned along its semi-major axis:

$$
\begin{aligned}
[A_1]\,[M_1(t)] &= [A_1]\,[M_1(\tau' + \tau_1)]\ , && (15a)\\
&= [A_1]\begin{bmatrix} \cos(\tau' + \tau_1) \\ \sin(\tau' + \tau_1) \end{bmatrix}, && (15b)\\
&= [A_1]\begin{bmatrix} \cos(\tau_1)\cos(\tau') - \sin(\tau_1)\sin(\tau') \\ \sin(\tau_1)\cos(\tau') + \cos(\tau_1)\sin(\tau') \end{bmatrix}, && (15c)\\
&= [A_1]\begin{bmatrix} \cos(\tau_1) - \sin(\tau_1) \\ \sin(\tau_1) + \cos(\tau_1) \end{bmatrix}\begin{bmatrix} \cos(\tau') \\ \sin(\tau') \end{bmatrix}, && (15d)\\
&= [A_1]\,[\psi_{\tau_1}]\,[M_1(\tau')]\ , && (15e)\\
&= [A_1']\,[M_1(\tau')]\ , && (15f)
\end{aligned}
$$

were $[\psi_{\tau_1}]$ is the rotation operator, rotating by an angle $\tau_1$ and $[A_1'] = [A_1]\,[\psi_{\tau_1}]$. The spatial angle at the shifted starting point $\varrho$ is given by

$$
\varrho(\nu) = \arctan\left(\frac{\gamma_1'}{\alpha_1'}\right) + \nu\pi\,. \tag{16}
$$

Again, a single, unique and correct solution for $\varrho$ can be obtained by using $\varrho = \mathrm{atan2}(\gamma_1', \alpha_1')$ instead. The starting point lies in quadrant III or IV when $\varrho < 0$. In that case, $\tau_1$ is modified as follows:

$$
\tau_1^\star = (\tau_1 + \pi)\ . \tag{17}
$$

Otherwise (when the starting point is already in quadrant I or II), $\tau_1^\star = \tau_1$.

The new EFA coefficients corrected for the starting point then become:

$$
\begin{bmatrix} \alpha_n^\star & \beta_n^\star \\ \gamma_n^\star & \delta_n^\star \end{bmatrix} = \begin{bmatrix} \alpha_n & \beta_n \\ \gamma_n & \delta_n \end{bmatrix}\begin{bmatrix} \cos(n\tau_1^\star) & -\sin(n\tau_1^\star) \\ \sin(n\tau_1^\star) & \cos(n\tau_1^\star) \end{bmatrix}. \tag{18}
$$

Finally, we ensure that the direction of contour approximation of the first harmonic is always counter-clockwise (i.e., that $r_1 \geq 0$, Equation 9). Besides removing redundancy by restricting the freedom of choice regarding the overall direction of contour approximation, this transformation also guarantees a unique correspondence between the properties of each subsequent harmonic and its contribution to the morphological features. When the direction of the first harmonic is clockwise ($r_1 < 0$), we therefore invert the direction of motion of all ellipses, maintaining thereby their inter-relationships. This can be done by running "time" backwards:

$$
\begin{aligned}
\begin{bmatrix} x(-t) \\ y(-t) \end{bmatrix} &= \begin{bmatrix} \alpha_n^\star & \beta_n^\star \\ \gamma_n^\star & \delta_n^\star \end{bmatrix}\begin{bmatrix} \cos\left(\frac{2n\pi(-t)}{T}\right) \\ \sin\left(\frac{2n\pi(-t)}{T}\right) \end{bmatrix}\\
&= \begin{bmatrix} \alpha_n^\star & \beta_n^\star \\ \gamma_n^\star & \delta_n^\star \end{bmatrix}\begin{bmatrix} \cos\left(\frac{2n\pi t}{T}\right) \\ -\sin\left(\frac{2n\pi t}{T}\right) \end{bmatrix}\\
&= \begin{bmatrix} \alpha_n^\star & -\beta_n^\star \\ \gamma_n^\star & -\delta_n^\star \end{bmatrix}\begin{bmatrix} \cos\left(\frac{2n\pi t}{T}\right) \\ \sin\left(\frac{2n\pi t}{T}\right) \end{bmatrix}.
\end{aligned} \tag{19}
$$

In short, whenever $r_1 < 0$, all indices $\beta_n^\star$ and $\delta_n^\star$ should be negated. After these steps, each unique cell contour is represented by a unique set of EFA coefficients. Note that the steps above do not alter the layout, nor do they rotate the shape. In certain study contexts, however, it might be desirable to rotate the contour itself, positioning



Development • Supplementary information

the semi-major axis, for example, to be parallel to the x-axis (or in any other preferred orientation). The details on how to perform those rotations can be found in Kuhl and Giardina (1982). Please note that unlike in their study, our subsequent analysis does not require such a cell contour realignment.

For simplicity of notation in the rest of the Supplementary Materials and Methods we refer to the $[A_n]$ matrix, which elements have been normalised regarding the starting point and direction of reconstruction of the first harmonic:

$$[A_n] \equiv \begin{bmatrix} a_n & b_n \\ c_n & d_n \end{bmatrix} \equiv \begin{bmatrix} \alpha_n^\star & \beta_n^\star \\ \gamma_n^\star & \delta_n^\star \end{bmatrix} . \tag{20}$$

After all possible sources of redundancy have been removed, the next step of the LOCO-EFA method is to split each elliptic harmonic into two counter-rotating circles.

### (2) Decompose each $n$th elliptic harmonic into two circles with opposite direction of rotation

In order to find the contribution of $n$th harmonic to a given morphological feature, we rewrite the $[A_n]$ matrices in Equation 10 such as to explicitly introduce the length of the semi-major and semi-minor axis of the $n$th ellipse ($\lambda_{1n}$ and $\lambda_{2n}$).

For this purpose, it is necessary to introduce both the temporal and the spatial rotation operator of each elliptic harmonic, given by $[\psi_{T_n}]$ and $[\psi_{S_n}]$, respectively. The temporal operator is defined as

$$[\psi_{T_n}] = \begin{bmatrix} \cos\phi_n & -\sin\phi_n \\ \sin\phi_n & \cos\phi_n \end{bmatrix} , \tag{21}$$

and the spatial operator is defined as

$$[\psi_{S_n}] = \begin{bmatrix} \cos\theta_n & -\sin\theta_n \\ \sin\theta_n & \cos\theta_n \end{bmatrix} , \tag{22}$$

where $\phi_n$ is the temporal angle (i.e., the time $\tau_n$ required to rotate to the semi-major axis) and $\theta_n$ the spatial angle (i.e., the angle of this position along the semi-major axis with the positive x-axis) (Figure S8A).

Equation 11 can be written as:

$$[X(t)] = [A_0] + \sum_{n=1}^{N} [\psi_{S_n}] [\psi_{S_n}]^{-1} [A_n] [\psi_{T_n}] [\psi_{T_n}]^{-1} [M_n(t)] , \tag{23}$$

given that $[\psi_{S_n}][\psi_{S_n}]^{-1}$and $[\psi_{T_n}] [\psi_{T_n}]^{-1}$ correspond to the identity matrix $[I]$.

Equation 11 can also be written in a form which directly highlights the contribution of the semi-major and semi-minor axis:

$$[X(t)] = [A_0] + \sum_{n=1}^{N} [\psi_{S_n}] [\Lambda_n] [\psi_{T_n}]^{-1} [M_n(t)] . \tag{24}$$

This equation can be understood as follows: for each mode, correctly position the starting point relative to the semi-major axis, transform the original circle into an ellipse, its semi-major axis along the x-axis and semi-minor axis along the y-axis, and finally rotate the ellipse to its correct position (see Figure S8B–D). Since both descriptions should



Development • Supplementary information

be equivalent, by combining Equation 23 and Equation 24, the $[\Lambda_n]$ matrix can be identified, with $\lambda_{1n}$ corresponding to the length of the semi-major axis, and the modulus of $\lambda_{2n}$ to the length of the semi-minor axis of the $n$th ellipse:

$$[\Lambda_n] = [\psi_{S_n}]^{-1} [A_n] [\psi_{T_n}] = \begin{bmatrix} \lambda_{1n} & 0 \\ 0 & \lambda_{2n} \end{bmatrix} . \tag{25}$$

Note that this process is similar to a singular value decomposition of $[A_n]$, with the difference that here $\lambda_{2n}$ (but not $\lambda_{1n}$) can be negative. The temporal angle $\phi_n$ corresponds, for each elliptic harmonic, to the scaled time $\tau_n$ to reach an extreme along the semi-major axis (Figure S8). Similarly to Equation 13, this corresponds to

$$\phi_n(\nu) = \frac{1}{2} \arctan\left(\frac{2(a_n b_n + c_n d_n)}{a_n^2 + c_n^2 - b_n^2 - d_n^2}\right) + \frac{\nu}{2}\pi . \tag{26}$$

Once again, using atan2 ensures that $\phi_n$ corresponds to the semi-major axis (see above). For the next steps of the analysis (see below) it is essential that $\phi_n$ corresponds to the temporal angle to reach the semi-major axis, not the semi-minor axis, hence usage of atan2 or any equivalent function which determines the quadrant of the return value is essential.

The spatial angle $\theta_n$ (see Figure S8) can be calculated after applying the temporal modification:

$$\theta_n(\nu) = \arctan \frac{c_n^{'}}{a_n^{'}} + \nu\pi , \tag{27}$$

where $c_n^{'}$ and $a_n^{'}$, are the new coefficients after the temporal transformation (i.e., after applying $[A_n] [\psi_{T_n}]$). Again, it is essential to use atan2 to ensure that $\phi_n$ and $\theta_n$ are both relative to the same extreme of the semi-major axis. It does not matter, however, which of the two extremes is being used, hence for this step no check is required regarding the quadrants. Applying Equation 25 then provides $\lambda_{1n}$ and $\lambda_{2n}$. Deriving a temporal and spatial angle relative to the semi-major axis and both being related to the same extreme guarantees that $\lambda_{1n} \geq 0$ (while $\lambda_{2n}$ can be positive or negative, depending on the rotation direction of the rotor) and $|\lambda_{1n}| \geq |\lambda_{2n}|$.

Using the above, the EFA (Equation 11) can be rewritten as:

$$\begin{bmatrix} x(t) \\ y(t) \end{bmatrix} = [A_0] + \sum_{n=1}^{N} \begin{bmatrix} \cos\theta_n & -\sin\theta_n \\ \sin\theta_n & \cos\theta_n \end{bmatrix} \begin{bmatrix} \lambda_{1n} & 0 \\ 0 & \lambda_{2n} \end{bmatrix} \begin{bmatrix} \cos\phi_n & \sin\phi_n \\ -\sin\phi_n & \cos\phi_n \end{bmatrix} \begin{bmatrix} \cos\left(\frac{2n\pi t}{T}\right) \\ \sin\left(\frac{2n\pi\tau t}{T}\right) \end{bmatrix} . \tag{28}$$

Written in this form, the contribution of each harmonic can easily be separated to correctly map to morphological feature number. The diagonal matrix containing the length of the semi-major and semi-minor axis of each $n$th mode can be decomposed into two diagonal matrices, each corresponding to circular orbits moving in opposite directions:

$$\begin{bmatrix} x(t) \\ y(t) \end{bmatrix} = [A_0] + \sum_{n=1}^{N} \begin{bmatrix} \cos\theta_n & -\sin\theta_n \\ \sin\theta_n & \cos\theta_n \end{bmatrix} \left( \begin{bmatrix} \lambda_{+_n} & 0 \\ 0 & \lambda_{+_n} \end{bmatrix} + \begin{bmatrix} \lambda_{-_n} & 0 \\ 0 & -\lambda_{-_n} \end{bmatrix} \right) \begin{bmatrix} \cos\phi_n & \sin\phi_n \\ -\sin\phi_n & \cos\phi_n \end{bmatrix} \begin{bmatrix} \cos\left(\frac{2n\pi t}{T}\right) \\ \sin\left(\frac{2n\pi t}{T}\right) \end{bmatrix} , \tag{29}$$



Development • Supplementary information

where $\lambda_{+_n}$ and $\lambda_{-_n}$ are the radii of those circles (Figure 2).

Summing up the diagonal matrices in Equation 29 yields

$$\begin{bmatrix} x(t) \\ y(t) \end{bmatrix}$$
$$= [A_0] + \sum_{n=1}^{N} \begin{bmatrix} \cos\theta_n & -\sin\theta_n \\ \sin\theta_n & \cos\theta_n \end{bmatrix} \begin{bmatrix} \lambda_{+_n} + \lambda_{-_n} & 0 \\ 0 & \lambda_{+_n} - \lambda_{-_n} \end{bmatrix} \begin{bmatrix} \cos\phi_n & \sin\phi_n \\ -\sin\phi_n & \cos\phi_n \end{bmatrix} \begin{bmatrix} \cos\left(\frac{2n\pi t}{T}\right) \\ \sin\left(\frac{2n\pi t}{T}\right) \end{bmatrix}, \tag{30}$$

in which the major and minor axes of each elliptic harmonic are

$$\lambda_{1_n} = \lambda_{+_n} + \lambda_{-_n}\,, \tag{31a}$$
$$\lambda_{2_n} = \lambda_{+_n} - \lambda_{-_n}\,, \tag{31b}$$

and hence the radii of each oppositely-rotating circle is given by

$$\lambda_{+_n} = (\lambda_{1_n} + \lambda_{2_n})/2\,, \tag{32a}$$
$$\lambda_{-_n} = (\lambda_{1_n} - \lambda_{2_n})/2\,. \tag{32b}$$

Given that $\lambda_{1n} \geq 0$ and $|\lambda_{1n}| \geq |\lambda_{2n}|$, $\lambda_{+_n}$ and $\lambda_{-_n}$ are always positive. To approximate the cell contour $(x(t), y(t))$ using the circles $\lambda_{+_n}$ and $\lambda_{-_n}$ requires completing the transformations using the spatial $(\theta_n)$ and temporal angle $(\phi_n)$ as calculated before, most clearly seen through the expression

$$[X(t)] = [A_0] + \sum_{n=1}^{N} [\psi_{S_n}]\,[\Lambda_{+_n} + \Lambda_{-_n}]\,[\psi_{T_n}]^{-1}\,[M_n(t)]\;. \tag{33}$$

The term $\Lambda_{+_n}$ presents the subset of the $n$th elliptic harmonic which is moving in the same direction as the first harmonic, therefore purely contributing to $n-1$ "lobes", i.e., shape features with periodicity $n-1$. In contrast, $\Lambda_{-_n}$ presents the subset moving in the opposite direction, purely contributing to $n+1$ "lobes" (shape features) only. Their contributions can be separated by writing:

$$[X(t)] = [A_0] + \sum_{n=1}^{N} [A_{+_n}]\,[M_n(t)] + \sum_{n=1}^{N} [A_{-_n}]\,[M_n(t)]\;, \tag{34}$$

where

$$[A_{j_n}] = [\psi_{S_n}]\,[\Lambda_{j_n}]\,[\psi_{T_n}]^{-1}\,, \quad \text{for } j = +, -\,. \tag{35}$$

This can be further simplified. Straightforwardly, $[\psi_{S_n}]\,[\Lambda_{+_n}] = [\psi_{S_n}]\,\lambda_{+_n}\,[I] = [\Lambda_{+_n}]\,[\psi_{S_n}]$. Regarding $[\Lambda_{+_n}]$,

$$[\psi_{S_n}]\,[\Lambda_{-_n}] = \begin{bmatrix} \cos\theta_n & -\sin\theta_n \\ \sin\theta_n & \cos\theta_n \end{bmatrix} \begin{bmatrix} \lambda_{-_n} & 0 \\ 0 & -\lambda_{-_n} \end{bmatrix}, \tag{36a}$$
$$= \begin{bmatrix} \lambda_{-_n}\cos\theta_n & +\lambda_{-_n}\sin\theta_n \\ \lambda_{-_n}\sin\theta_n & -\lambda_{-_n}\cos\theta_n \end{bmatrix}, \tag{36b}$$
$$= \begin{bmatrix} \lambda_{-_n} & 0 \\ 0 & -\lambda_{-_n} \end{bmatrix} \begin{bmatrix} \cos\theta_n & \sin\theta_n \\ -\sin\theta_n & \cos\theta_n \end{bmatrix}, \tag{36c}$$
$$= \begin{bmatrix} \lambda_{-_n} & 0 \\ 0 & -\lambda_{-_n} \end{bmatrix} \begin{bmatrix} \cos(-\theta_n) & -\sin(-\theta_n) \\ \sin(-\theta_n) & \cos(-\theta_n) \end{bmatrix}, \tag{36d}$$
$$= [\Lambda_{-_n}]\,[\psi_{S_n}]^{-1}\;. \tag{36e}$$



Development • Supplementary information

Thus,

$$[A_{+n}] = [\Lambda_{+n}] [\psi_{S_n}] [\psi_{T_n}]^{-1} , \tag{37a}$$
$$[A_{-n}] = [\Lambda_{-n}] [\psi_{S_n}]^{-1} [\psi_{T_n}]^{-1} . \tag{37b}$$

Hence, introducing $\zeta_{+n} = \theta_n - \phi_n$ and $\zeta_{-n} = -\theta_n - \phi_n$, Equation 35 can be written as

$$[A_{jn}] = [\Lambda_{jn}] \left[\psi_{\zeta_{jn}}\right] , \quad \text{for } j = +, - , \tag{38}$$

which corresponds to

$$\begin{aligned} [A_{jn}] &= \begin{bmatrix} a_{jn} & b_{jn} \\ c_{jn} & d_{jn} \end{bmatrix} \\ &= \begin{bmatrix} \lambda_{jn} & 0 \\ 0 & j\lambda_{jn} \end{bmatrix} \begin{bmatrix} \cos\zeta_{jn} & -\sin\zeta_{jn} \\ \sin\zeta_{jn} & \cos\zeta_{jn} \end{bmatrix} , \quad \text{for } j = +, - . \end{aligned} \tag{39}$$

We next label those matrices with respect to their lobe contribution instead of their EFA mode. To make the distinction, we here use subscript $n$ to indicate the EFA mode, and subscript $l$ to indicate the LOCO-EFA mode. In general, $[A_{+l}] = \left[A_{+n+1}\right]$ and $[A_{-l}] = \left[A_{-n-1}\right]$. There are, however, a few exceptions, see Figure S4:

1) The overall offset of the contour is not solely given by $[A_0]$. An additional contribution to the offset is coming from $[A_{+n=2}]$. Note, however, that the contribution from $[A_{+n=2}]$ is in fact not a perfect offset to the contour, but also causes a kidney bean-shaped distortion to the contour, its deviation from a pure offset becoming more pronounced when the contribution of this mode relative to the overall contour size is larger (see Figure S4B).

2) The overall circular shape of the contour (i.e., LOCO-mode 1) receives solely a contribution from $[A_{+n=1}]$ itself, i.e., $[A_{+l=1}] = [A_{+n=1}]$.

3) When $N$ EFA modes are taken into account, then LOCO-mode $N$ only receives a contribution from $\left[A_{-n=N-1}\right]$.

4) Likewise, when taking $N$ EFA modes into account, there is still a contribution to LOCO-mode $L = N + 1$, solely coming from $[A_{-n=N}]$.

Defining $[M_{+l}] = [M_{n+1}]$ and $[M_{-l}] = [M_{n-1}]$, with the exceptions $[M_{+l=0}] = [M_{n=2}]$ and $[M_{+l=1}] = [M_{n=1}]$, then the same shape approximation can be achieved through LOCO-EFA as through EFA:

$$\begin{aligned} [X(t)] = [A_0] &+ [A_{+l=0}] [M_{+l=0}(t)] + [A_{+l=1}] [M_{+l=1}(t)] \\ &+ \sum_{l=2}^{L-2} [A_{+l}] [M_{+l}(t)] + \sum_{l=2}^{L} [A_{-l}] [M_{-l}(t)] . \end{aligned} \tag{40}$$

Equation 40 can be used to reconstruct the shape up to a certain $L$ number, requiring EFA coefficients up to mode $L + 1$:

$$[X_{L=1}(t)] = [A_0] + [A_{+l=0}] [M_{+l=0}(t)] + [A_{+l=1}] [M_{+l=1}(t)] , \tag{41a}$$

$$[X_{L\geq 2}(t)] = [A_0] + [A_{+l=0}] [M_{+l=0}(t)] + [A_{+l=1}] [M_{+l=1}(t)] + \sum_{l=2}^{L} [A_{+l}] [M_{+l}(t)] + \sum_{l=2}^{L} [A_{-l}] [M_{-l}(t)] . \tag{41b}$$



Development • Supplementary information

Each LOCO-EFA mode can be fully described by the combination of the radii and the starting positions of both circles. The radii are, as previously defined, $\lambda_{+_l} = \lambda_{+_{n+1}}$ and $\lambda_{-_l} = \lambda_{-_{n-1}}$, with the exceptions $\lambda_{+_{l=0}} = \lambda_{+_{n=2}}$ and $\lambda_{-_{l=0}} = 0$, and $\lambda_{+_{l=1}} = \lambda_{+_{n=1}}$ and $\lambda_{-_{l=1}} = 0$. The starting points of both circles are defined as $\zeta_{+_l} = \zeta_{+_{n+1}}$ and $\zeta_{-_l} = \zeta_{-_{n-1}}$, again with the exceptions $\zeta_{+_{l=0}} = \zeta_{+_{n=2}}$ and $\zeta_{+_{l=0}} = \zeta_{+_{n=2}}$. Together this gives a set of four coefficients $\mathcal{L}_l = (\lambda_{+_l}, \lambda_{-_l}, \zeta_{+_l}, \zeta_{-_l})$ that fully capture each LOCO-EFA mode and allow for a full reconstitution of the original shape:

$$\begin{aligned}[X(t)] = [A_0] + [\Lambda_{+_{l=0}}] \left[\psi_{\zeta_{+_{l=0}}}\right] [M_{+_{l=0}}(t)] + [\Lambda_{+_{l=1}}] \left[\psi_{\zeta_{+_{l=1}}}\right] [M_{+_{l=1}}(t)] \\ + \sum_{l=2}^{L-2} [\Lambda_{+_l}] \left[\psi_{\zeta_{+_l}}\right] [M_{+_l}(t)] + \sum_{l=2}^{L} [\Lambda_{-_l}] \left[\psi_{\zeta_{-_l}}\right] [M_{-_l}(t)] \,. \end{aligned} \tag{42}$$

The exceptions for the lowest modes, as depicted in Figure S4A might appear counterintuitive. Their particular repairing is however a direct consequence of how we have chosen to define mode 0 and mode 1. Our choice was driven by seeking to stay more in line with classical EFA. As in EFA, we have defined mode 0 as the offset of the contour with respect to the underlying coordinate system, and therefore mode 1 as the radius of the circle, the most basic capturing of the shape itself. LOCO-EFA modes, in contrast, should be viewed as corresponding to a specific number of perturbations along that circle (for its mathematical derivation see further details in the next session). Hence, an alternative choice of definitions could have been to denominate the radius of the circle mode 0. The unidirectional perturbation of the circle as provided by EFA modes 0 and 2 could be interpreted as a 'single lobe', and could therefore be denominated mode 1, etc. Although such a defendable renaming of LOCO-EFA modes would make Figure S4A free of any apparent exceptions in mode reassignment, we preferred to maintain the definitions as here presented, for sake of clarity. The reason being that, although it introduces exceptions that carefully have to be taken care of, the chosen denomination captures, alike EFA, within mode 0 the non-oscillatory "DC" contour offset. Renaming that to mode 1 would in our eyes be more unnatural than to simply reassign modes as is currently done.

In the next section we derive how from those values a single amplitude value can be found, the $L_l$ contribution.

### (3) $L_l$ amplitude contributions

In this section, we first show that the contribution to the shape of the two counter-rotating circles of a specific LOCO-EFA mode is not simply determined by the radii of those circles, but also depends on the relative position of the starting points. We then derive a heuristic which optimally captures its contribution, determining as well the limits of this approximation. To illustrate how a difference between the starting points $\zeta_{+_l}$ and $\zeta_{-_l}$ of mode $l$ can affect the generated amplitude, we first plot the contribution of $\mathcal{L}_3 = (\lambda_{+_3}, \lambda_{-_3}, \zeta_{+_3}, \zeta_{-_3})$ superimposed on $\mathcal{L}_1 = (1, 0, 0, 0)$, i.e., superimposed on the unit circle starting at zero degrees. As long as $\lambda_{+_3}$, $\lambda_{-_3}$ are not too large, a single amplitude value $a$ (which is the amplitude of deviation from the unit circle) can be observed for any phase $\omega$; peak amplitude values $A$ occur for specific phases $\Omega$ (as illustrated in Figure S9I). Figure S9A–C depict the shape contribution of the negative rotor (green), positive rotor (red) and their summed contribution (orange), as a function of the phase $\omega$, for $\mathcal{L}_3 = (0.15, 0.15, 0, 0)$. This scenario illustrates that even though the negative rotor makes two sidereal rotations while the positive rotor makes four sidereal rotations, the amplitude



Development • Supplementary information

pattern that they generate not only has a period three in both cases, as argued throughout the paper, but also perfectly match one another regarding the phases at which the peaks and troughs in the amplitude pattern are reached. The summed contribution (Figure S9C) therefore indeed yields a peak amplitude exactly equal to $\lambda_{+3} + \lambda_{-3}$. In contrast, when both rotors are exactly out-of-phase ($\mathcal{L}_3 = (0.15, 0.15, 0, \pi)$, Figure S9D–F), the peaks and troughs are exactly out-of-phase as well, and the patterns almost (but not totally) cancel each other out (Figure S9F). To determine the effective contribution of a LOCO-EFA mode we therefore have to determine the phase at which each rotor reaches its peak amplitude.

The angles $\Omega_+, \Omega_-$ at which the positive and negative rotor reach their peak amplitude can be calculated straightforwardly. They occur when the phase of the rotor itself is equivalent to the overall phase generated by $\mathcal{L}_1$ (as illustrated in Figure S9G). The phase of $\mathcal{L}_1$ starts at $\zeta_{+1}$, while the phase of the positive rotor starts at $\zeta_{+l}$ (illustrated in Figure S9K). When the pattern is laid down, the phase of the positive rotor changes $(l+1)$ times faster than the phase of $\mathcal{L}_1$ (Equation 42). Thus, regarding the phase at peak amplitude it holds that

$$\Omega_{+l} = (l+1)\,(\Omega_{+l} - \zeta_{+1}) + \zeta_{+l} + 2\pi\nu\,, \tag{43a}$$
$$-l\Omega_{+l} = \zeta_{+l} - (l+1)\zeta_{+1} + 2\pi\nu\,, \tag{43b}$$
$$\Omega_{+l} = \zeta_{+1} + \frac{\zeta_{+1} - \zeta_{+l}}{l} + \frac{2\pi}{l}\nu\,. \tag{43c}$$

where $\nu$ is any integer, and the values $\nu = 0, 1, \ldots, l-1$ provide the complete set of phases at which peak amplitude is reached. Likewise, the negative rotor rotates $(l-1)$ times faster than $\mathcal{L}_1$ and in the opposite direction, hence starting at $-\zeta_{-l}$ (see Figure S9J). Regarding the phase at peak amplitude it therefore holds that

$$\Omega_{-l} = -(l-1)\,(\Omega_{+l} - \zeta_{+1}) - \zeta_{-l} + 2\pi\nu\,, \tag{44a}$$
$$l\Omega_{-l} = -\zeta_{-l} + (l-1)\zeta_{+1} + 2\pi\nu\,, \tag{44b}$$
$$\Omega_{-l} = \zeta_{+1} - \frac{\zeta_{+1} + \zeta_{-l}}{l} + \frac{2\pi}{l}\nu\,. \tag{44c}$$

To assess the summed amplitude contribution, we next fit the amplitude pattern laid down by each rotor to a sine wave, exactly matching both the peak amplitude and the phase at which the peak amplitude is reached, and then sum those two contributions:

$$a_{+l} = \lambda_{+l}\cos\left(l\omega + \zeta_{+1} - (l+1)\zeta_{+1}\right)\,, \tag{45a}$$
$$a_{-l} = \lambda_{-l}\cos\left(l\omega + \zeta_{-l} - (l-1)\zeta_{+1}\right)\,, \tag{45b}$$
$$a_{+l} + a_{-l} = \lambda_{+l}\cos\left(l\omega + \zeta_{+l} - (l+1)\zeta_{+1}\right) + \lambda_{-l}\cos\left(l\omega + \zeta_{-l} - (l-1)\zeta_{+1}\right)\,. \tag{45c}$$

Equation 45a exactly matches the peak amplitude and its phase of the positive rotor, as derived in Equation 43, while Equation 45b exactly matches the peak amplitude and its phase of the negative rotor, as derived in Equation 44. While peak amplitude and phase match perfectly, Figure S9G, H illustrate that for not too large values of $\lambda_{+l}$, $\lambda_{-l}$ also the rest of the pattern presents a close match.

Using standard trigonometry, the summed amplitude can be written as



Development • Supplementary information

$$a_{+_l} + a_{-_l} = a_l = \sqrt{\lambda_{+_l}^2 + \lambda_{-_l}^2 + 2\lambda_{+_l}\lambda_{-_l}\cos\left(\zeta_{+_l} - \zeta_{-_l} - 2\zeta_{+_1}\right)}\cos\left(l\omega + \zeta_l\right) \; , \qquad (46a)$$

where

$$\zeta_l = \mathrm{atan2}\left(\lambda_{+_l}\sin\left(\zeta_{+_1} - (l+1)\zeta_{+_1}\right) + \lambda_{-_l}\sin\left(\zeta_{-_l} - (l-1)\zeta_{+_1}\right), \lambda_{+_l}\cos\left(\zeta_{+_1} - (l+1)\zeta_{+_1}\right) + \lambda_{-_l}\cos\left(\zeta_{-_l} - (l-1)\zeta_{+_1}\right)\right) . \qquad (46b)$$

Figure S9I illustrates that for not too large values of $\lambda_{+_l}$, $\lambda_{-_l}$ this expression provides a close match to the pattern generated by the $\mathcal{L}_l$ mode, here illustrated using both a different phase and a different amplitude for both rotors. Using the equation above, the amplitude or contribution of mode $l$ can hence be defined as

$$L_l = \sqrt{\lambda_{+_l}^2 + \lambda_{-_l}^2 + 2\lambda_{+_l}\lambda_{-_l}\cos\left(\zeta_{+_l} - \zeta_{-_l} - 2\zeta_{+_1}\right)} . \qquad (47)$$

While above we have performed a more formal derivation, a more intuitive way to understand the amplitude contribution of the positive and negative rotor combined, is in terms of how much the amplitude contributions of both rotors are out-of-phase with each other. The most straightforward moment to determine their phase shift is at the initial point of drawing the cell's outline. As illustrated in Figure S9J, K, the phase shift regarding the amplitude contribution of the negative rotor is given by $\zeta_{+_1} + \zeta_{-_l}$, and of the positive rotor by $\zeta_{+_l} - \zeta_{+_1}$. To understand the graphical argument for the negative rotor case, one has to realise that the negative rotor rotates clockwise, and hence its starting angle is $-\zeta_{-_l}$ (for the positive rotor it is simply $\zeta_{+_l}$), while for the same reason the phase shift between initial amplitude and maximum amplitude has to be calculated clockwise (instead of standard counterclockwise, as done for the positive rotor), again as shown in Figure S9J, K. Plotting the phase shift and strength of both amplitude contributions as vectors and then adding them up then provides the above equation for $L_l$ (as is depicted in Figure 2C).

Note that the pattern can never be perfectly described by a sine wave, which is why four parameters are needed to describe each mode, rather than the two required by Equation 46. Even when following Equation 46 the two waves cancel each other out, this is not exactly the case, as seen in Figure S9F. There is therefore no additional level of redundancy, as the usage of the $L_l$ numbers might suggest, even when the value of $L_l = 0$. When the amplitude of a rotor becomes larger, the generated pattern starts to deviate from a sine wave (Figure S9J, K), and their summed pattern from Equation 46 (Figure S9L). For simple closed contours (which cell outlines should always be), the deviations cannot be very large, since otherwise the contour becomes non-simple (i.e., crosses itself). This confinement in deviations for cell outlines allows us to base a significant part of our analysis on $L_l$ values.

Moreover, we observe that the positive rotor generates protrusions that are flatter than a sine wave (Figure S9B, J), whereas the negative rotor generates lobes that are pointier (Figure S9A, K). The extent of "flatness" or "pointedness" of each mode can be determined by the proportion $w_{+_l} = \lambda_{+_l}/(\lambda_{+_l} + \lambda_{-_l})$ and $w_{-_l} = 1 - w_{+_l}$, respectively; a useful additional measure if needed.

The method presented here cannot be straightforwardly extended to 3D. The essential issue is that this whole analysis hinges on a parametrisation which moves along a directed curve. As no directed curve can be constructed to represent a cell outline in 3D, there is no simple extension from our 2D LOCO-EFA to 3D.



Development • Supplementary information

## $XOR$ measurement

LOCO-EFA can be applied to any closed-curve cell outline defined by a series of connected points. There are no requirements regarding the number of points or the distance between them, nor do their positions have to be integer or positive values. Nevertheless, the cell outlines that will be analysed with our method are generally derived from segmentation of microscopy images. Many segmentation methods are available, with most of them assigning each pixel within a microscopy image to a specific cell domain.

In the paper we show that, besides directly looking at the $L_n$ values, a highly informative analysis is to capture the quality of cell shape reconstruction when only a certain number of modes are taken into account. This is done by applying an EXCLUSIVE OR ($XOR$) between the segmented cell and reconstructed cell. The natural choice for the grid on which the $XOR$ is performed is therefore equivalent to the microscopy image itself, at the resolution the image has been acquired and segmentation performed, as is done for all the experimental images analysed in this study. In such a case, the cell contour, i.e., the edge of the segmented cell, takes up integer values (when expressed in grid points), with the distance between points being either 1 (when the cell edge moves horizontally or vertically) or $\sqrt{2}$ (when the cell edge moves diagonally). While the input of the LOCO-EFA analysis is a set of discrete points, the output that it provides is a continuous curve. The LOCO-EFA provides an infinite series of modes, passing increasingly closer to the original set of discrete points $(x_i, y_i)$ at parametrised time $t_i$ when more modes are taken into account. (For a derivation of the predicted bound on error as a function of mode number for the classical EFA, see (Kuhl and Giardina, 1982).) To reconstruct from that curve a cell shape on a grid therefore requires two steps: 1) Determine the grid points at which the curve passes at each time $t_i$. Please note that the time series $t_i$ has to correspond to the "time" passed while drawing the discrete chain of original contour points, as discussed below Equation 5, and hence will be a time series containing irregular intervals (of either 1 or $\sqrt{2}$). The reason is that by only evaluating the continuous curve at those time points, for sufficiently many modes the curve is guaranteed to lie within the grid point defining the original cell outline. In between those time points, however, the curve could very well cross through grid points that do not belong to the cell outline, especially when the cell outline involves a diagonal step. 2) Only in the case that the next reconstructed contour point lies beyond the Moore neighbourhood of the previous contour point, iteratively reduce the time steps between these two reconstructions (deviating from the specific time points as defined above), in order to acquire on the grid a contiguous chain of pixels defining the cell outline. Doing so allows for flood filling at the end to obtain the complete cell shape rather than just the cell outline, and to then apply an $XOR$ with the original segmented image.

LOCO-EFA provides infinitely many modes, for which it is at forehand not defined where a required cut-off should be placed, since the approximation becomes asymptotically more precise. The $XOR$ analysis, however, provides an algorithmic and objective cut-off for any LOCO-EFA data analysis, not only the $XOR$ analysis. When the reconstruction is performed as explained above, then for a sufficiently large, but finite number of modes each individual contour grid point will be correctly reconstructed, which leads to the $XOR$ value becoming zero. Adding additional terms will only change the reconstructed outline at a sub-pixel resolution, i.e., at a resolution that is higher than the original microscopy image. Obviously, the latter cannot be meaningful in any possible way. Using the $XOR$ reaching zero therefore provides a natural cut-off to truncate the



Development • Supplementary information

series, as higher-mode contributions can only be informative provided the $XOR$ is still non-zero, but cannot be informative beyond that point. In our experience, this point is usually reached between 20 and 200 modes, but will be cell-complexity and image-resolution dependent. Note that additional information acquired outside of the method could justify earlier cut-offs, for example when there is information available regarding the quality of segmentation.

## Final remarks

To summarise, the LOCO-EFA method consists in: 1) eliminating degeneracies in the EFA coefficients; 2) decomposing each elliptic harmonic into two circles rotating in opposite directions ($\lambda_{+n}$ and $\lambda_{-n}$) and therefore contributing to $n-1$ and $n+1$ number of lobes (more generally, morphological features); and 3) calculating the offset between starting points of these two circles derived from each ellipse to estimate the amplitude of the $L_l$th lobe contribution.

To eliminate cell area effects (for example, when looking at shape diversity within a cell populations), it might also be desirable to normalise for cell size by dividing each $L_n$ value by the square root of the cell area. This then provides a complete description of the number of lobes and their amplitude, which can be used to characterise and quantify intrinsic cell shape properties, irrespective of cell area, spacing between sampling points, and rotations or inversions of the cell (Figure S10A, B). Changes in the image resolution, however can of course affect the fine-grained information retrieval, but only if the resolution becomes very low (Figure S10C, D).

# Supporting Figures

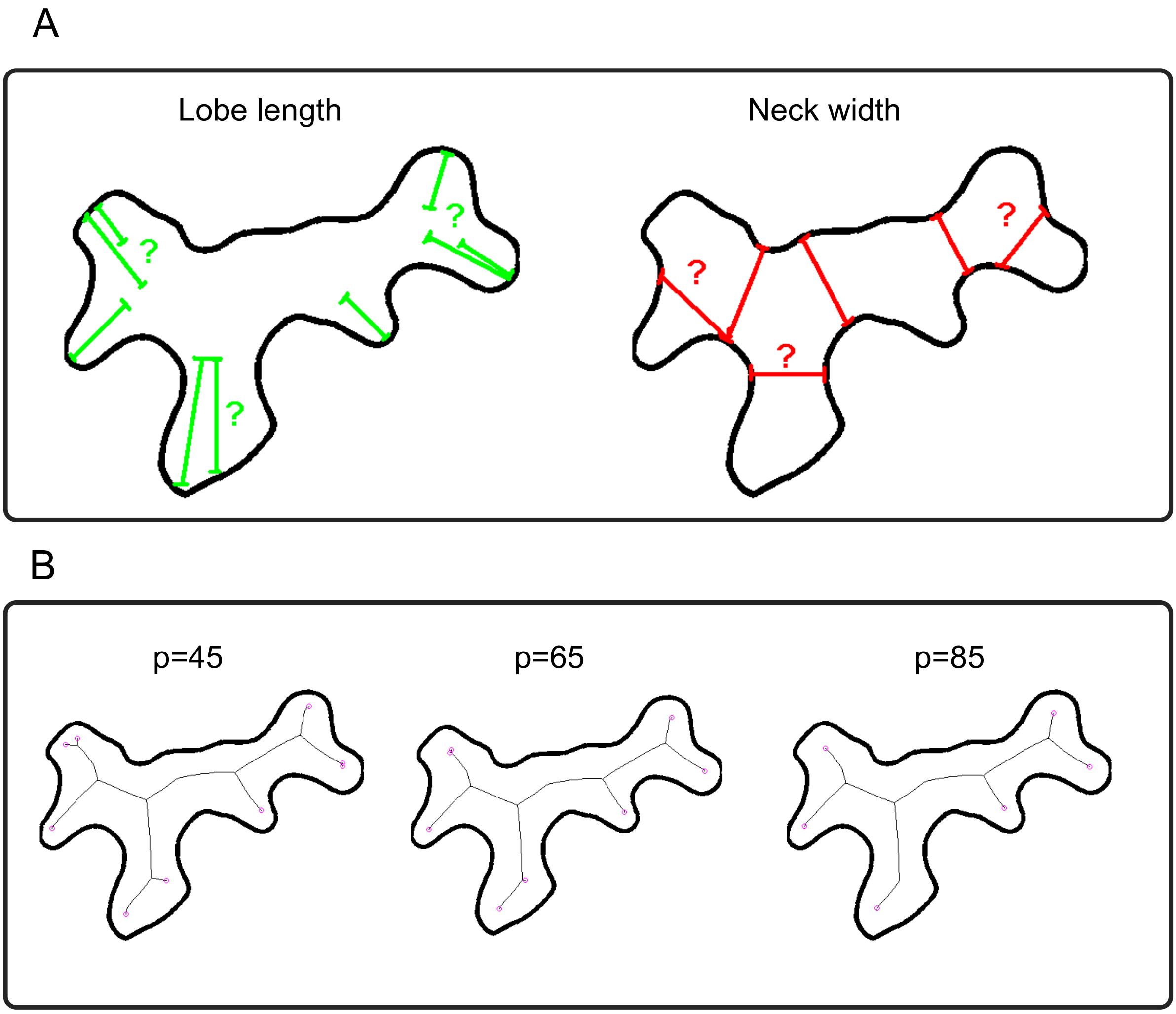


Figure S1: **Subjective choices involved in neck width and lobe length determination and Skeletonisation.** (A) Neck width and lobe length depend on human criteria for both identifying and quantifying such structures, as indicated by the question-marks. (B) Both the number of skeleton end-points and the length of the branches strongly depend on the parameter settings used for the skeletonisation algorithm. Here, parameter $p$ (see material and Methods) was set to 45 (left), 65 (middle), or 85 (right). For those values, the number of predicted lobes varies between 6 and 8.



Development • Supplementary information

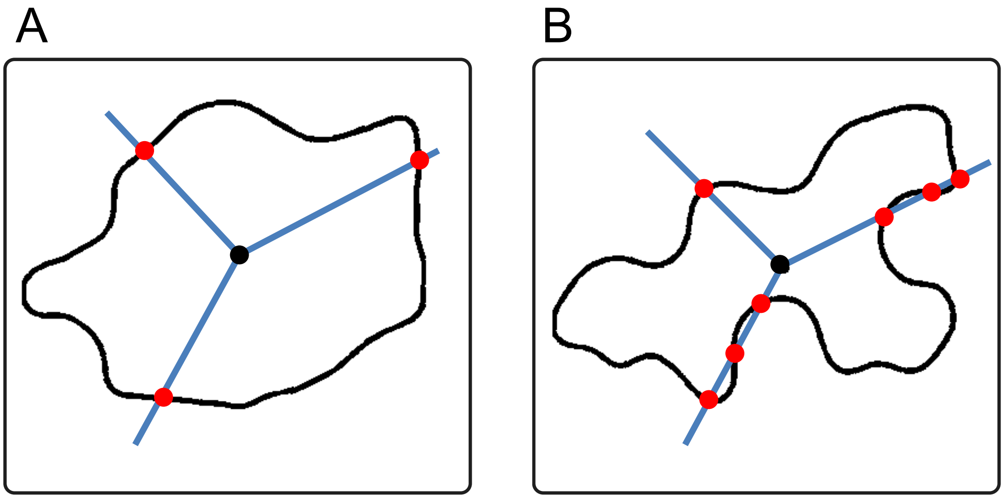


Figure S2: **Holomorphic and non-holomorphic shapes**. (A) In holomorphic shapes, all radii starting from the centroid intersect the outline only once. (B) In non-holomorphic shapes, some radii intersect more than once. Very few pavement cells have a holomorphic shape, the majority presenting highly complex non-holomorphic outlines. The outline of non-holomorphic shapes cannot be represented as a function of the angle, precluding, for example, standard Fourier analysis.



Development • Supplementary information

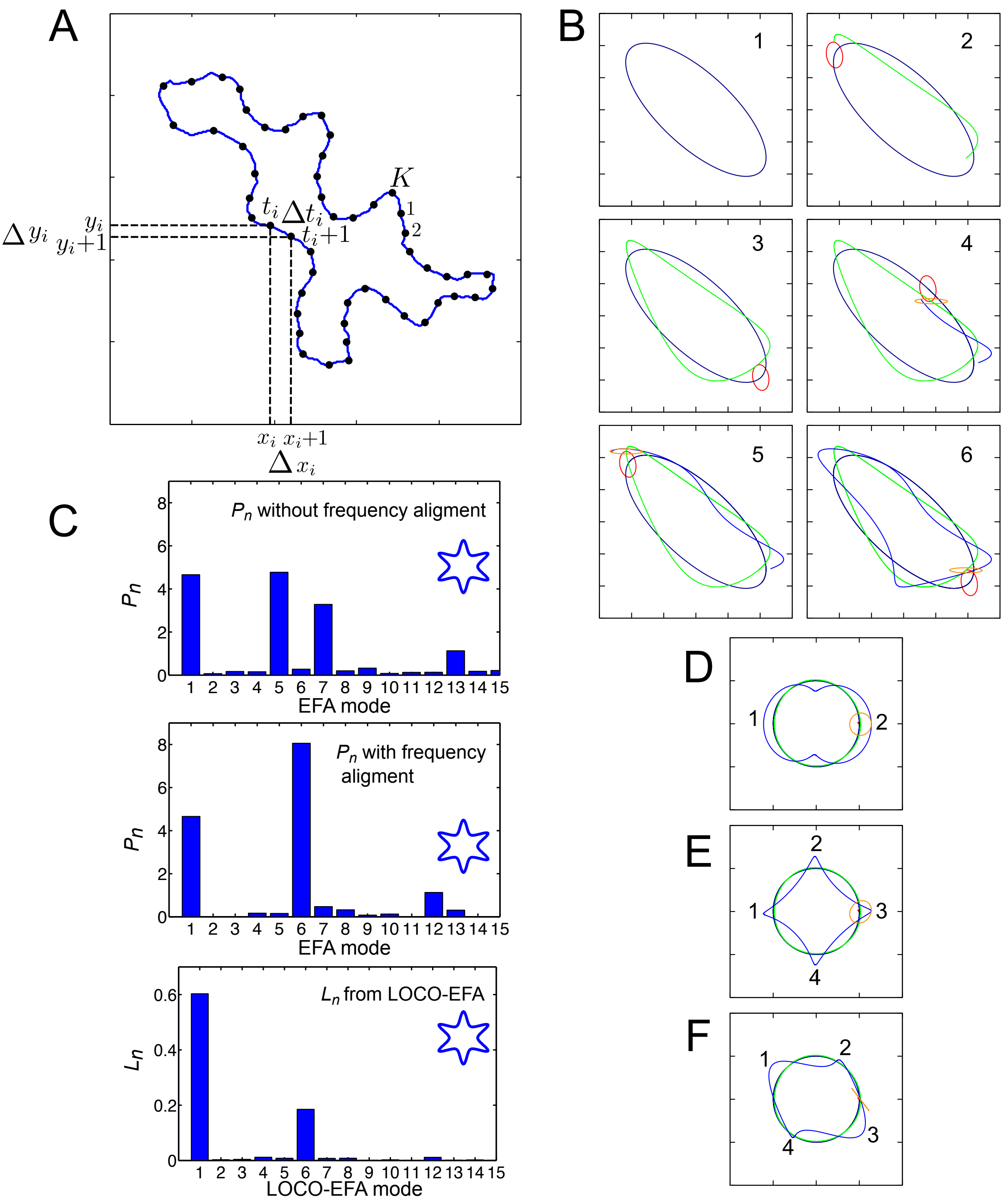


Figure S3

Development • Supplementary information

Figure S3: **Cell outline reconstruction using EFA and LOCO-EFA.** (A) Calculating the EFA coefficients from a cell outline. The discrete chain of contour points can be positioned arbitrarily (for example, the points do not have to be associated to an underlying grid). Also, the distances between points can be arbitrarily long. (B) Sequential approximation of the cell's contour. The first harmonic forms an elliptic shape (1, blue). The second harmonic describes an elliptic orbit (2–6, red), orbiting twice while moving around the first harmonic (2, 3). Their summed trajectories are shown in green. The third elliptic harmonic (4–6, orange) orbits thrice while moving around this summed trajectory (4–6). The summation of the first three elliptic harmonics is shown in blue. See Movie 1 for this dynamical reconstitution of the cell contour. (C) The EFA coefficients cannot be directly linked to shape features, here shown through the power contribution of each harmonic ($P_n$) (upper panel): the main $P_n$ contributions of a six-pointed star-like shape (shown as an inset) come from the 5th and 7th harmonic. To align the coefficients to actual shape features, Diaz et al. (1990) proposed to shift the contribution of each harmonic to either $n+1$ or $n-1$, depending on the rotation direction of each individual harmonic (middle panel). This brings the main shape contributor and the actual number of shape features in alignment to each other. This method, however, does not always hold (see F), and moreover generates a range of spurious contributions from a large set of different modes, which is a notorious issue that hampers analysis of complex shapes using standard EFA (Haines and Crampton, 2000). The LOCO-EFA method correctly aligns the shape assessment and the real shape features (lower panel), without generating any additional spurious contributions. Moreover, unlike the other methods, the values correctly represent the amplitude of the shape features. (D–F) Contours (shown in blue), generated from the first (green) and third EFA harmonic (orange) only. The number of morphological protrusions (lobes) specified by the $n$th EFA mode is affected, but not fully determined, by its rotation direction. The heuristic rule proposed by Diaz et al. (1990) states that if the first harmonic and the $n$th harmonic rotate in the same direction, a contour is generated with $n-1$ protrusions, while if their rotation direction is opposite, the contour will contain $n+1$ lobes. This rule, however, is only correct when the elliptic harmonic has a circular shape (D, E), in which case indeed overall shapes are generated with either 2 (D) or 4 lobes (E), simply dependent on the rotation direction with respect to the first mode. When, however, the elliptic harmonic has a higher eccentricity (F), the final shape can have $n+1$ protrusions (4 lobes) even though the rotation direction of the first and third harmonic are the same. In D–F the determinant of the first harmonic is -1.0002 (i.e., rotating clockwise), while the determinants of the third harmonics are $-6.554 \cdot 10^{-2}$ (clockwise), $6.446 \cdot 10^{-2}$ (counter-clockwise), and $-1.19 \cdot 10^{-4}$ (clockwise), respectively. The heuristic fails as a consequence of each single EFA mode actually contributing to two different spatial modes. See also Movie 2–4.

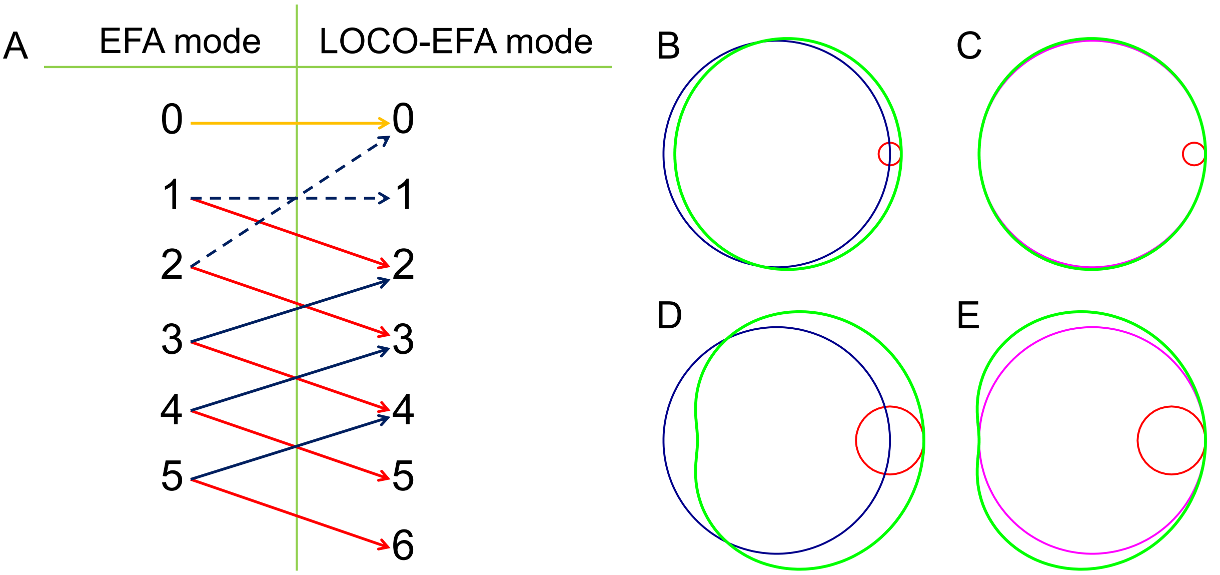


Figure S4: **Schematic mapping between EFA modes and LOCO-EFA modes**. (A) Each $n$th EFA mode contributes to both $n+1$ and $n-1$ morphological periodicities. The red arrows represent the contributions of the $n$th EFA mode to $n+1$ protrusions, due to the clockwise rotations of the circular harmonics $\lambda_{-_{n+1}}$. The blue arrows indicate the contributions to $n-1$ protrusions, due to the counter-clockwise rotations of the circular harmonics $\lambda_{+_{n-1}}$. A few exceptions apply: The second EFA mode contributes to a shift in the positioning of the layout, i.e., to LOCO-EFA mode 0 ($\lambda_{+_0}$, blue and dashed line), rather than to the overall size of the layout, as might have been expected. The first EFA mode contributes to the overall circular size of the layout ($\lambda_{+_1}$, blue and dashed line), rather than to a shift in the positioning of the layout, as might have been expected. The zeroth EFA mode only contributes to a shift in the positioning of the layout (yellow line). Finally, the two highest LOCO-EFA modes have incomplete contributions, given any cutoff in the number of EFA modes. (B–E) The contribution $\lambda_{+_0}$ is not simply an offset of the contour, but also involves a kidney bean-shaped distortion, more pronounced for larger contributions. Mode $\lambda_{+_1}$ (the circular mode) is shown in blue; mode $\lambda_{+_0}$ in red; their summation in green; and the predicted shape if the contribution were purely an offset in magenta. (B) $\lambda_{+_0}$ being 10% of $\lambda_{+_1}$, effectively resulting in just an offset to the shape outline (C). (D, E) $\lambda_{+_0}$ being 30% of $\lambda_{+_1}$, resulting in a clear kidney bean-shaped distortion of the shape.



Development • Supplementary information

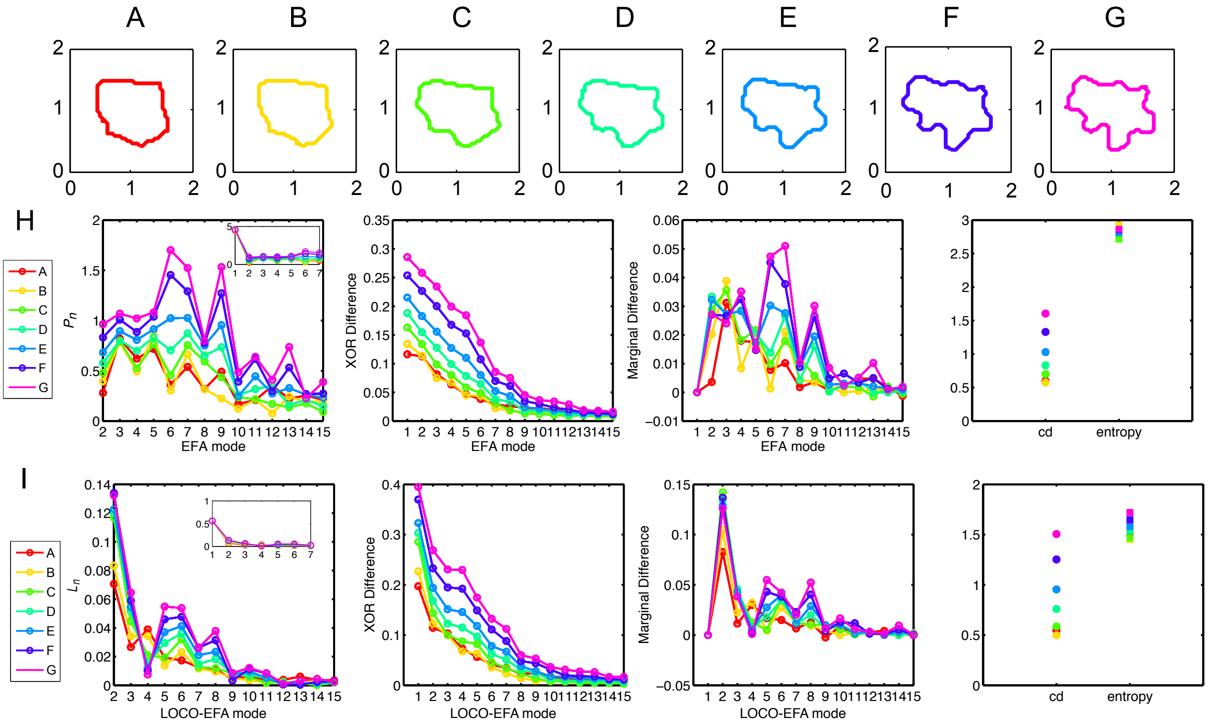


Figure S5: **Additional example of LOCO-EFA metrics on a cell changing its shape over time**. (A–G) Sequence of a tracked pavement cell growing over time with normalised area. (H) $P_n$, $XOR$ difference and marginal difference profiles, cumulative difference and entropy using EFA. (I) $L_n$, $XOR$ difference and marginal difference profiles, cumulative difference and entropy using LOCO-EFA. See also the first example presented in Figure 4 in the main text.

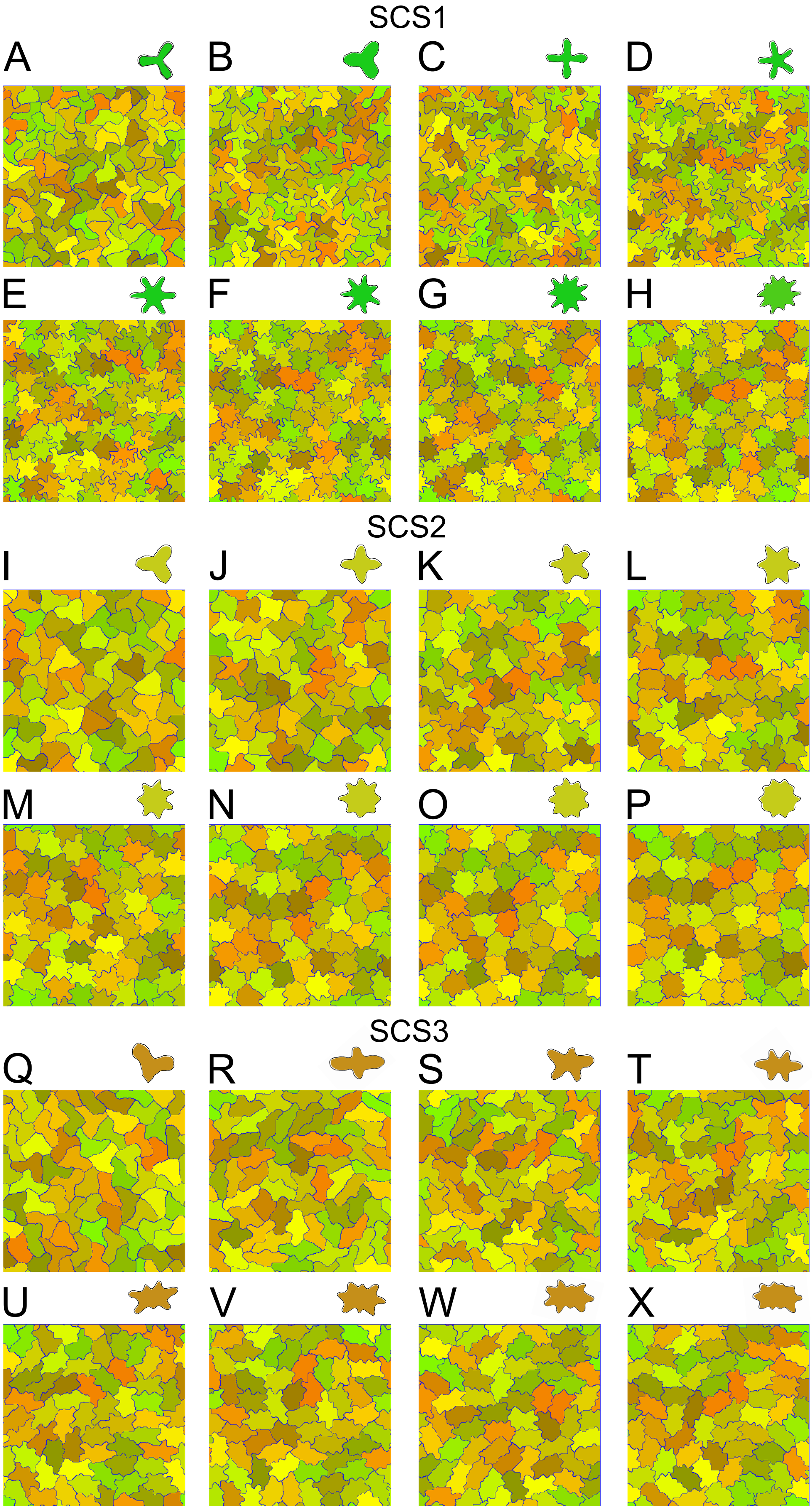


Figure S6



Development • Supplementary information

Figure S6: **Confluent *in silico* cell populations, simulated with three types of specified cell shapes (SCSs) and different number of specified lobes.** Parameters for the different specified cell shapes are given in Table S1. (A–H) Cells with large protrusions (Specified Cell Shapes 1, SCS1), with specified lobe number increasing from 3 (A) to 10 (H); (I–P) Cells with small protrusions (SCS2), with specified lobe number increasing from 3 (I) to 10 (P); (Q–X) Elongated cells (SCS3), with specified lobe number increasing from 3 (Q) to 10 (X). Specified cell shapes, as resulting from single-cell simulations, are shown above each panel. Cells are randomly coloured. See also Figure 5 and Figure S7.

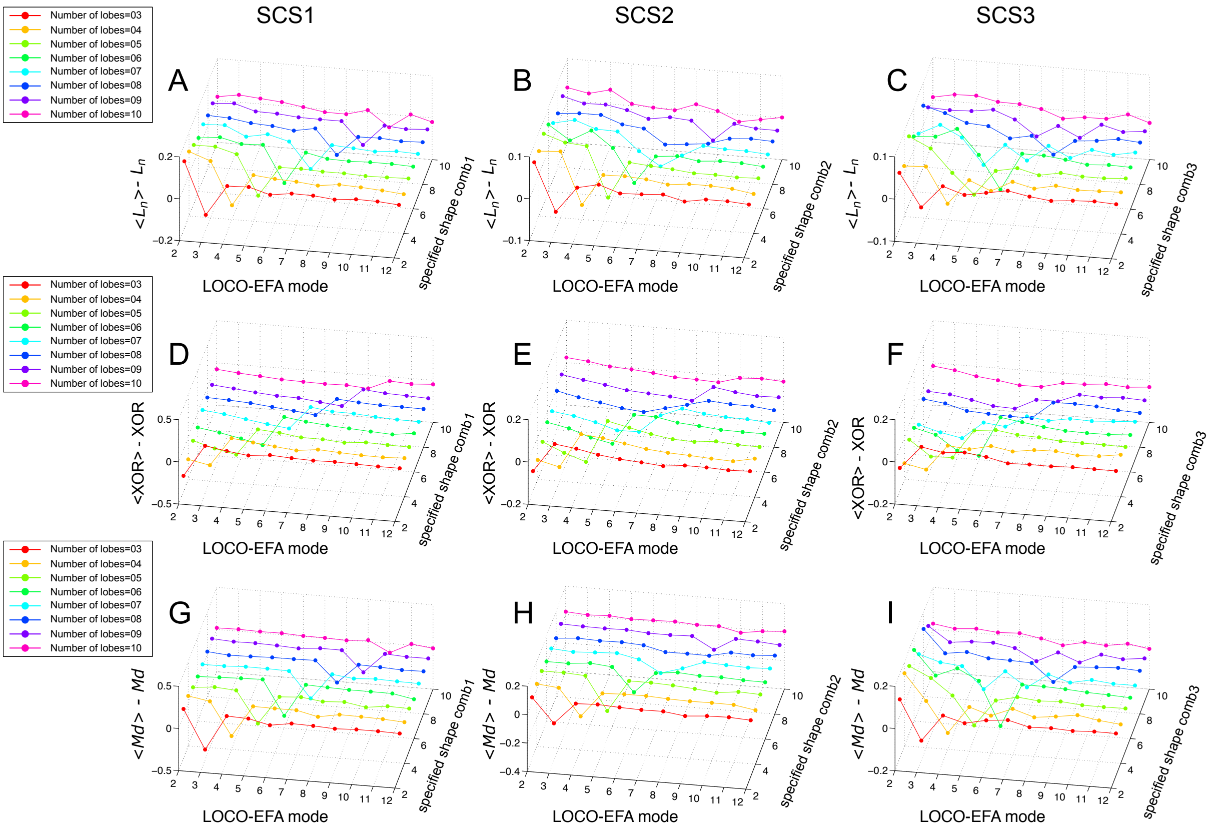


Figure S7: **Divergence between specified cell shapes and resultant population-level cell shape diversity within confluent *in silico* cell populations.** The mismatch is visualised as the difference in LOCO-EFA-derived measures between the average for confluent population simulations and its value for single cell simulations. Three types of specified cell shapes (SCSs) were simulated, as indicated for each column, and parametrised in Table S1. Lobe numbers vary from 3 to 10, as indicated for each row and depicted in Figure S6. (A–C) Difference between the average $L_n$ ($\langle L_n \rangle$) values of the cells of the simulated populations and that of a single simulated cell. (D–F) Difference between average $XOR$ ($\langle XOR \rangle$) of the cells of the simulated population and that of a single simulated cell. (G–I) Same as in (A–C, D–F), but now for marginal difference (Md). In all cases the specified cell shape becomes less pronounced in the confluent population simulations (indicated by negative values that all measures yield at the given specified lobe number), while the cells present an increased shape diversity and complexity (seen by the broad flanking regions with positive values in the profiles, indicating a large range of modes that contribute to the shapes). These effects are present regardless of the number of specified lobes or the combination of parameters used, but becomes less pronounced at higher lobe numbers. See also Figure 5. Each depicted line is based upon averaging all cells within three independent tissue simulations using the same shape descriptors; each simulated tissue contains in between 42 and 70 *in silico* cells.

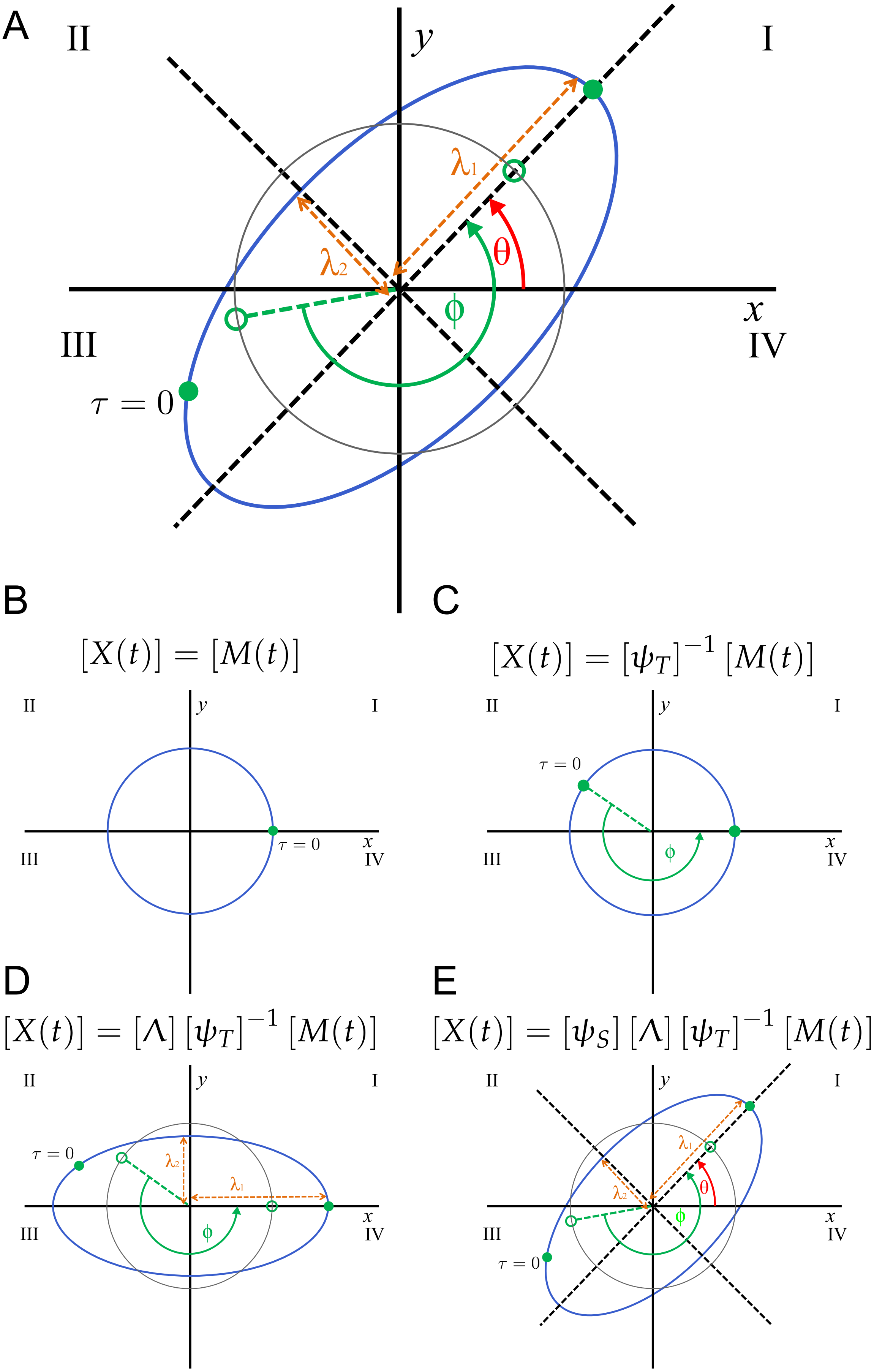


Figure S8

Development • Supplementary information

Figure S8: **Temporal and spatial transformations required to calculate the precise contribution of each EFA mode.** (A) The blue ellipse depicts an elliptic harmonic, given by $[X(t)] = [A]\,[M(t)]$, while the grey circle depicts the unit circle, given by $[X(t)] = [M(t)]$. The temporal angle $\phi$ is the scaled time required to move along an elliptic harmonic from the starting point at $\tau = 0$ to one extreme along the semi-major axis (green filled circles). The angle $\phi$ cannot be trivially derived from the spatial position at $\tau = 0$, requiring first an effective projection upon the unit circle (green open circles). The spatial angle $\theta$ is the inclination of the elliptic harmonic. After applying the appropriate spatial and temporal transformations to the EFA coefficients using this geometrical interpretation, the semi-major and semi-minor axis, $\lambda_1$ and $\lambda_2$, can be straightforwardly obtained. To eliminate multiple representations of the same outline, the starting point of the first harmonic is specifically positioned at the extreme along the semi-major axis which lies in either quadrant I or II, given by temporal angle $\tau_1^\star$ (Equation 18). In contrast, all other temporal angles $\phi_n$ used, while also positioned along the semi-major axis, are not confined to quadrant I or II. (B–E) Visual interpretation of Equation 24 as a step-wise construction of the elliptic harmonic. The matrix $[M(t)]$ corresponds to the unit circle (B). For each mode, first the starting point relative to the semi-major axis is correctly positioned (C); then the original circle is transformed into an ellipse, its semi-major axis along the x-axis and semi-minor axis along the y-axis (D); and finally, the ellipse is rotated to its correct position (E). See further details in the Supplementary Materials and Methods.



Development • Supplementary information

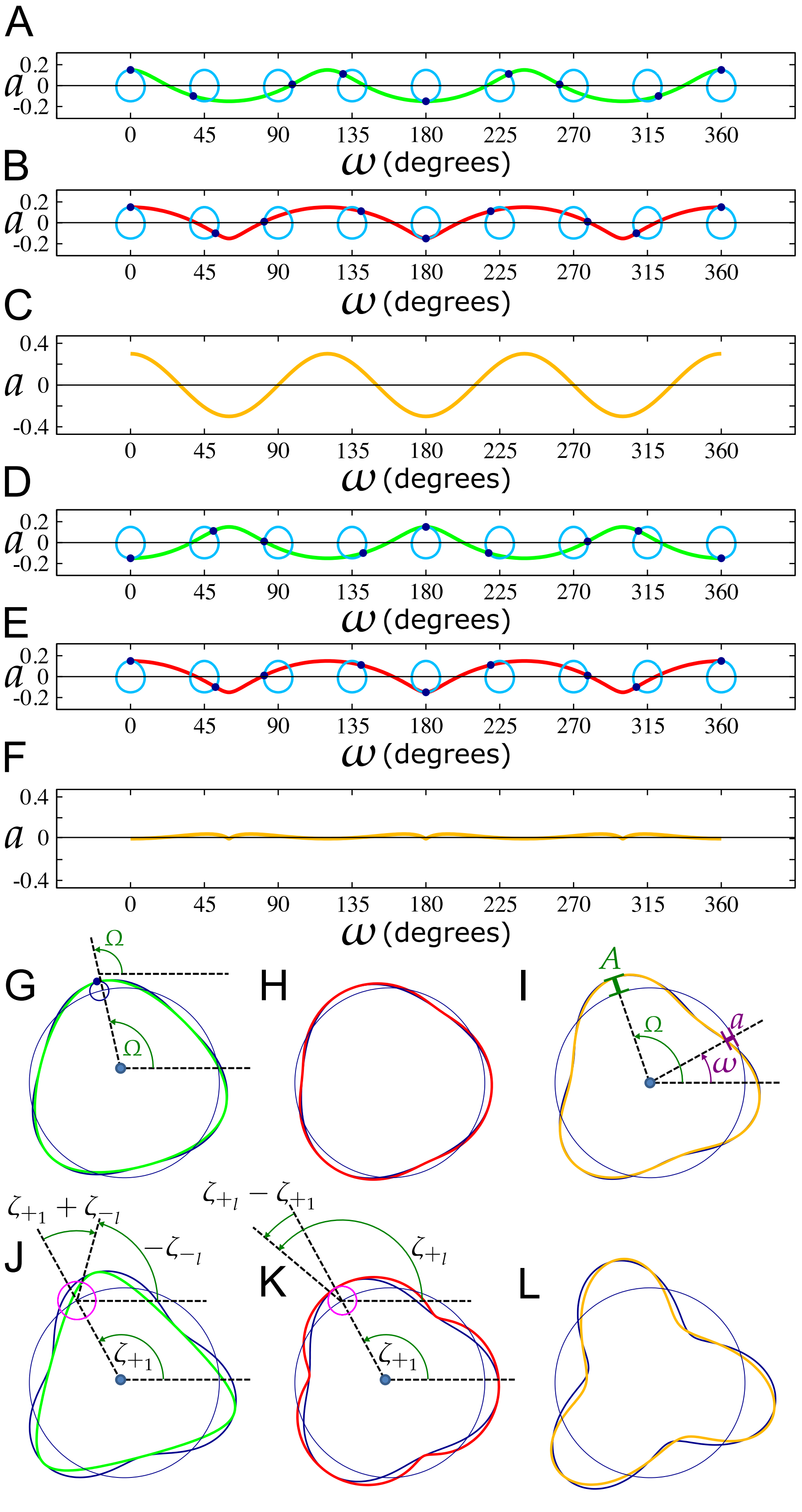


Figure S9



Development • Supplementary information

Figure S9: **Impact of LOCO-EFA starting points $\zeta_{+l}$ and $\zeta_{-l}$ on the amplitude of the reconstructed shape.** All panels show $\mathcal{L}_3 = (\lambda_{+3}, \lambda_{-3}, \zeta_{+3}, \zeta_{-3})$ superimposed on $\mathcal{L}_1 = (1, 0, \zeta_{+1}, 0)$, i.e., the unit circle. In (A–F), $\mathcal{L}_1 = (1, 0, 0, 0)$. (A) Amplitude $a$ as a function of phase $\omega$ (as depicted in I) for the negative rotor only ($\mathcal{L}_3 = (0, 0.15, 0, 0)$), shown in green. For several phases also the rotor (light blue) itself is drawn. Note that by plotting the rotor in the $(\omega, a)$ plane, the circles are slightly deformed. (B) Same for the positive rotor only ($\mathcal{L}_3 = (0.15, 0, 0, 0)$), in red. (C) Same for both rotors superimposed ($\mathcal{L}_3 = (0.15, 0.15, 0, 0)$), in orange. (D–F) Alike (A–C), but for an out-of-phase starting angle of the negative rotor. (D) $\mathcal{L}_3 = (0, 0.15, 0, \pi)$, in green; (E) $\mathcal{L}_3 = (0.15, 0, 0, 0)$, in red; (F) $\mathcal{L}_3 = (0.15, 0.15, 0, \pi)$, in orange. In (G–L), $\mathcal{L}_1 = \left(1, 0, \frac{2}{3}\pi, 0\right)$. (G) Pattern generated by the negative rotor only ($\mathcal{L}_3 = \left(0, 0.1, 0, -\frac{5}{12}\pi\right)$), shown in green, compared to the equation $a_{-l} = \lambda_{-l} \cos\left(l\omega + \zeta_{-l} - (l-1)\zeta_{+1}\right)$ (Equation 45b), in dark blue. They present a close match. (H) Same for the positive rotor only ($\mathcal{L}_3 = \left(0.075, 0, \frac{3}{4}\pi, 0\right)$), shown in red, compared to $a_{+l} = \lambda_{+l} \cos\left(l\omega + \zeta_{+l} - (l+1)\zeta_{+1}\right)$ (Equation 45a), show in dark blue, again presenting a close match. (I) Same for both rotors superimposed ($\mathcal{L}_3 = \left(0.075, 0.1, \frac{3}{4}\pi, -\frac{5}{12}\pi\right)$), shown in orange, and $a_l = \sqrt{\lambda_{+l}^2 + \lambda_{-l}^2 + 2\lambda_{+l}\lambda_{-l}\cos\left(\zeta_{+l} - \zeta_{-l} - 2\zeta_{+1}\right)} \cos\left(l\omega + \zeta_l\right)$ (Equation 46), show in dark blue, again presenting a close match. Panel (G) also illustrates that at peak amplitude the phase of the main and subrotor are equal; panel (I) also illustrates the concepts amplitude ($a$); peak amplitude ($A$); phase ($\omega$); and phase at peak amplitude ($\Omega$). (J–L) Alike (G–I), but for larger amplitude. (J) $\mathcal{L}_3 = \left(0, 0.2, 0, -\frac{5}{12}\pi\right)$, in green; (K) ($\mathcal{L}_3 = \left(0.15, 0, \frac{3}{4}\pi, 0\right)$, in red; (L) $\mathcal{L}_3 = \left(0.15, 0.2, \frac{3}{4}\pi, -\frac{5}{12}\pi\right)$, in orange. Panel (J) and (K) also illustrate how the initial phase shifts with respect to the amplitude contribution of the negative and positive rotor depend on $\zeta_{+1}$, and on $\zeta_{-l}$ and $\zeta_{+l}$, respectively. They are given by $\zeta_{+1} + \zeta_{-l}$ for the negative rotor and by $\zeta_{+l} - \zeta_{+1}$ for the positive rotor.



Development • Supplementary information

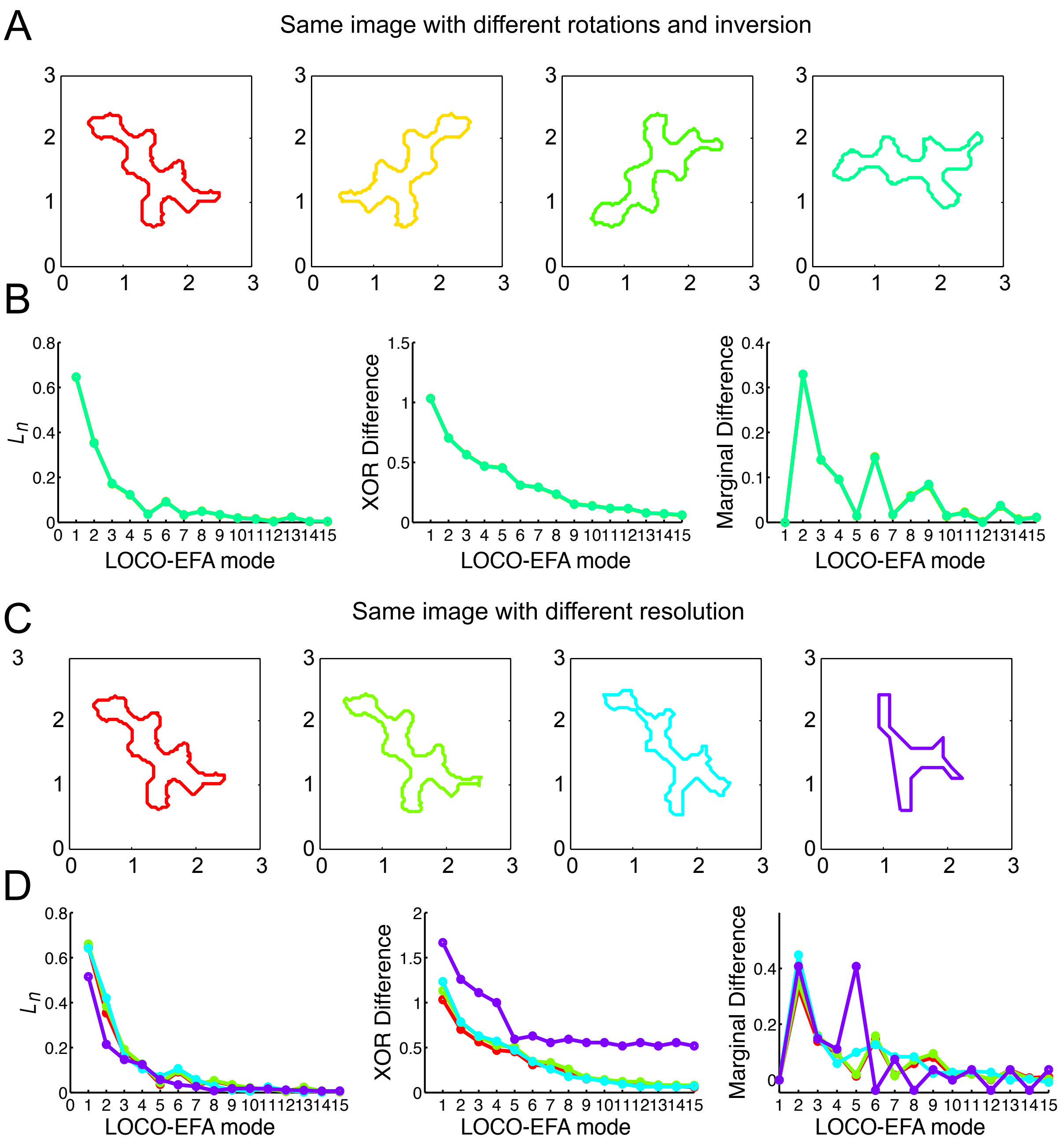


Figure S10: **LOCO-EFA is invariant to image rotation or mirroring, nor sensitive to image resolution.** (A) The cell outline of an experimentally observed cell was mirrored along the y-axis and/or rotated over different angles, after which LOCO-EFA was applied to each image separately. (B) The $L_n$ values and other derived metrics were invariant to those transformations. (C) The resolution of the original image was reduced, such that the number of contour points decreased from 1104 to 253, 108 and 27 (from left to right, respectively). (D) The $L_n$ numbers and the associated metrics only deviated from the high-resolution values when the resolution was very low and the cell outline was clearly deviating from original cell outline. This in contrast to the skeletonisation method, for which even marginal resolution reductions can cause large deviations in the outcome.



Development • Supplementary information

# Supporting Table

Table S1: Three types of specified cell shapes (SCS) as used in Figure S6 and Figure S7.

| | SCS1 | SCS2 | SCS3 |
|---|---|---|---|
| Target Area | 858 | 1167 | 1197 |
| Pointedness | 6912 | 5328 | 5207 |
| Number of lobes | 3–10 | 3–10 | 3–10 |
| Roundness | 382 | 518 | 434 |
| Elongation | 4 | 28 | 5927 |

# C code of LOCO-EFA method

Code written in C which applies the full procedure to calculate the $\mathcal{L}_l$ and $L_l$ values to any file containing contour coordinates. For each computational step, it is indicated where the relevant mathematical details can be found in the Supplementary Materials and Methods. We have intentionally kept the code as bare as possible, without for example any graphical interface, to allow it to be trivially compiled and run on any platform. Details regarding compilation and execution can be found in the header of the file.

Click here to Download C code of LOCO-EFA method

# Outline example for code

Contour data of the cell presented in Figure 3S, Figure S3A and Figure S10. This file, or any other file containing contour data, can be used as an input for the program.

Click here to Download Outline example for code



Development • Supplementary information

# Supporting Movies

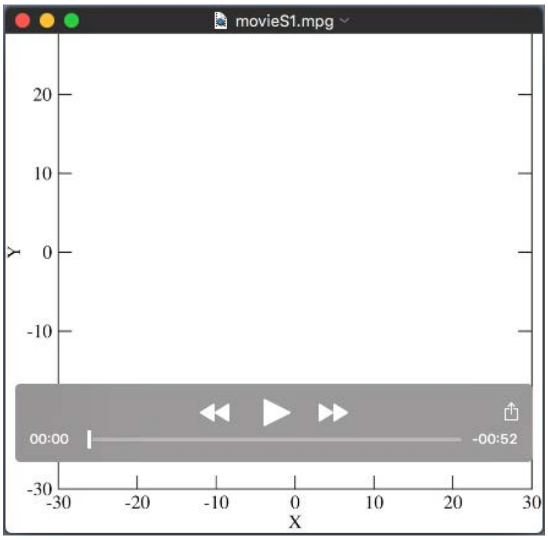


Movie 1: **Approximation of a closed contour using Elliptic Fourier Analysis.** A given two-dimensional shape can be approximated using EFA by summing $n$ elliptic harmonics as follows: each $n$th elliptic harmonic traces $n$ clockwise or counter-clockwise revolutions while moving around the previous elliptic harmonic.

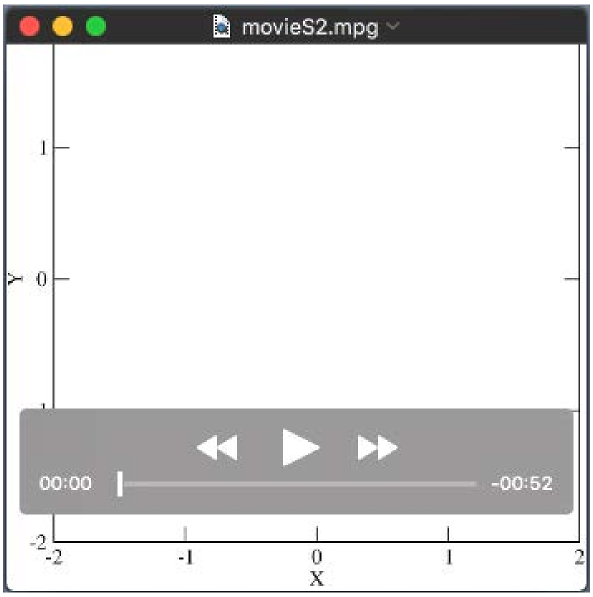


Movie 2: **Direction of rotation opposite to the first harmonic ellipse.** EFA mode 3 generating a shape with 4 features, its rotation direction opposite to the rotation direction of the first harmonic.

Development • Supplementary information

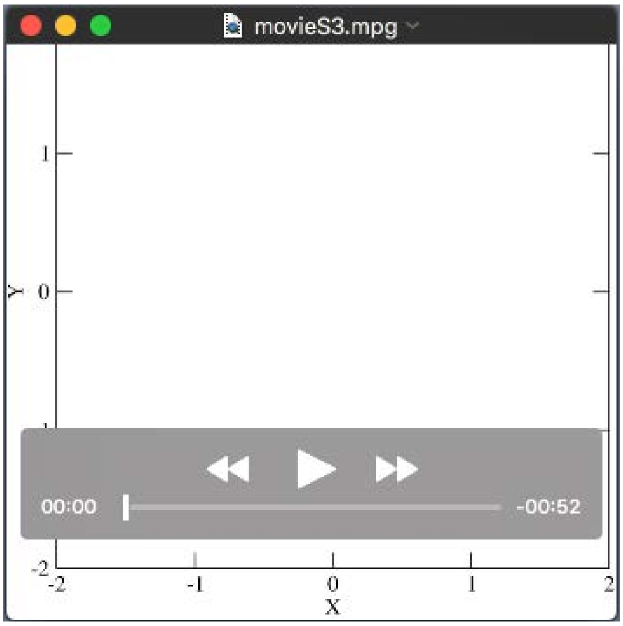


Movie 3: **Direction of rotation same as the first elliptic harmonic.** EFA mode 3 generating a shape with 2 features, its rotation direction the same as the rotation direction of the first harmonic.

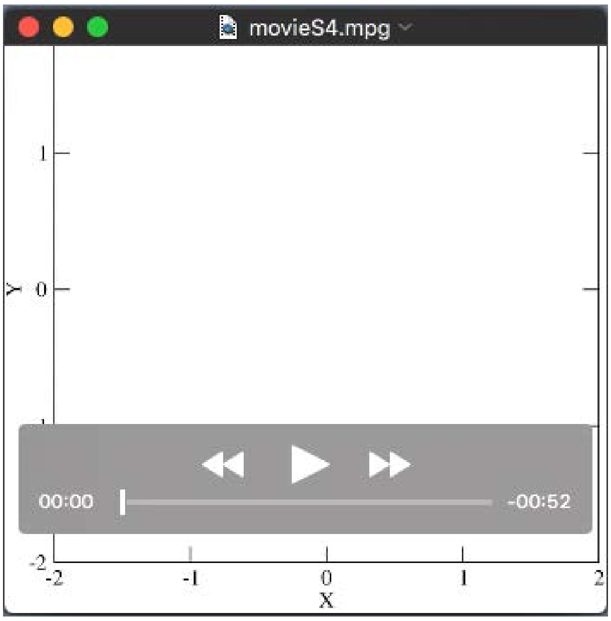


Movie 4: **Exception of the rule regarding rotation direction.** If the eccentricity of an elliptical harmonic is very high, the number of generated lobes does not follow the rule-of-thumb proposed by Diaz et al. (1990). Here, EFA mode 3 generates a shape with 4 features, although its rotation direction is the same as the rotation direction of the first harmonic.

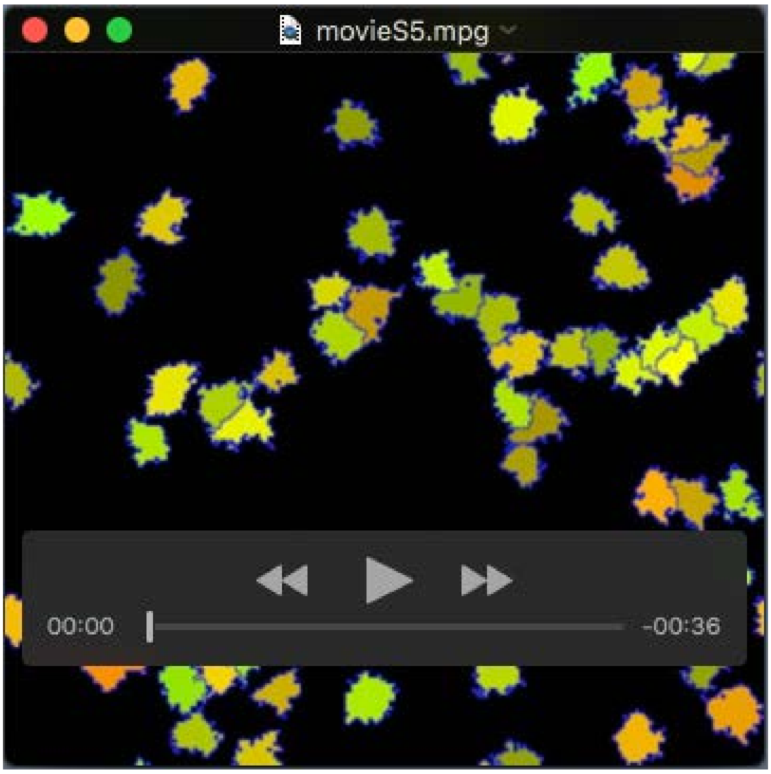


Movie 5: **Example of an *in silico* simulation of a population of cells with more complex specified shapes.** CPM simulation of multi-lobed cells take up pavement-like cell shapes when they are allowed to interact with their neighbours within a confluent tissue.

Development • Supplementary information

Development • Supplementary information

# Supporting References

**D. H. Chitwood, R. Kumar, L. R. Headland, A. Ranjan, M. F. Covington, Y. Ichihashi, D. Fulop, J. M. Jiménez-Gómez, J. Peng, J. N. Maloof and N. R. Sinha**. A quantitative genetic basis for leaf morphology in a set of precisely defined tomato introgression lines. *Plant Cell*, 25(7):2465–2481 (Jul 2013). doi:10.1105/tpc.113.112391. URL http://dx.doi.org/10.1105/tpc.113.112391.

**G. Diaz, D. Quacci and C. Dell'Orbo**. Recognition of cell surface modulation by elliptic Fourier analysis. *Comput. Methods Programs. Biomed.*, 31(1):57–62 (Jan 1990).

**M. Frieß and M. Baylac**. Exploring artificial cranial deformation using elliptic Fourier analysis of Procrustes aligned outlines. *Am. J. Phys. Anthropol.*, 122(1):11–22 (Sep 2003). doi:10.1002/ajpa.10286. URL http://dx.doi.org/10.1002/ajpa.10286.

**A. J. Haines and J. S. Crampton**. Improvements to the method of Fourier shape analysis as applied in morphometric studies. *Palaeontology*, 43(4):765–783 (2000).

**H. Iwata, K. Ebana, Y. Uga, T. Hayashi and J.-L. Jannink**. Genome-wide association study of grain shape variation among *Oryza sativa* L. germplasms based on elliptic Fourier analysis. *Mol. Breeding*, 25:203–215 (2010).

**H. Iwata, S. Niikura, S. Matsuura, Y. Takano and Y. Ukai**. Evaluation of variation of root shape of Japanese radish (*Raphanus sativus* L.) based on image analysis using elliptic Fourier descriptors. *Euphytica*, 102:143–149 (1998).

**F. P. Kuhl and C. R. Giardina**. Elliptic Fourier features of a closed contour. *Comput. Gr. Image Process.*, 18:236–258 (1982).

**J. C. Neto, G. E. Meyer, D. D. Jones and A. K. Samal**. Plant species identification using Elliptic Fourier leaf shape analysis. *Comput. Electron. Agric.*, 50:121–134 (2006).

**Z. Pincus and J. A. Theriot**. Comparison of quantitative methods for cell-shape analysis. *J. Microsc.*, 227(Pt 2):140–156 (Aug 2007). doi:10.1111/j.1365-2818.2007.01799.x. URL http://dx.doi.org/10.1111/j.1365-2818.2007.01799.x.

**M. Schmittbuhl, B. Allenbach, J.-M. Le Minor and A. Schaaf**. Elliptical descriptors: some simplified morphometric parameters for the quantification of complex outlines. *Math. Geol.*, 35:853–871 (2003).

**Y. Yoshioka, H. Iwata, R. Ohsawa and S. Ninomiya**. Quantitative evaluation of the petal shape variation in *Primula sieboldii* caused by breeding process in the last 300 years. *Heredity*, 94(6):657–663 (Jun 2005). doi:10.1038/sj.hdy.6800678. URL http://dx.doi.org/10.1038/sj.hdy.6800678.
